# Hierarchical Protofilament Intertwining Rules the Formation of Mixed‐Curvature Amyloid Polymorphs

**DOI:** 10.1002/advs.202402740

**Published:** 2024-06-20

**Authors:** Jiangtao Zhou, Salvatore Assenza, Meltem Tatli, Jiawen Tian, Ioana M. Ilie, Eugene L. Starostin, Amedeo Caflisch, Tuomas P. J. Knowles, Giovanni Dietler, Francesco S. Ruggeri, Henning Stahlberg, Sergey K. Sekatskii, Raffaele Mezzenga

**Affiliations:** ^1^ Laboratory of Physics of Living Matter, Institute of Physics Ecole Polytechnique Fédérale de Lausanne (EPFL) Lausanne CH‐1015 Switzerland; ^2^ Department of Health Sciences and Technology ETH Zurich Zurich Switzerland; ^3^ Departamento de Física Teórica de la Materia Condensada Universidad Autónoma de Madrid Madrid 28049 Spain; ^4^ Condensed Matter Physics Center (IFIMAC) Universidad Autónoma de Madrid Madrid 28049 Spain; ^5^ Instituto Nicolás Cabrera Universidad Autónoma de Madrid Madrid 28049 Spain; ^6^ Laboratory of Biological Electron Microscopy Institute of Physics SB EPFL and Dep. of Fund. Microbiol. Faculty of Biology and Medicine UNIL Rt. de la Sorge Lausanne 1015 Switzerland; ^7^ van't Hoff Institute for Molecular Sciences University of Amsterdam P.O. Box 94157 Amsterdam 1090 GD The Netherlands; ^8^ Amsterdam Center for Multiscale Modeling (ACMM) University of Amsterdam P.O. Box 94157 Amsterdam 1090 GD The Netherlands; ^9^ Department of Civil Environmental & Geomatic Engineering University College London Gower Street London WC1E 6BT UK; ^10^ Department of Biochemistry University of Zürich Zürich CH‐8057 Switzerland; ^11^ Department of Chemistry University of Cambridge Lensfield Road Cambridge CB2 1EW UK; ^12^ Laboratory of Organic Chemistry Wageningen University & Research Stippeneng 4 Wageningen 6703 WE The Netherlands; ^13^ Physical Chemistry and Soft Matter Wageningen University & Research Stippeneng 4 Wageningen 6703 WE The Netherlands; ^14^ Department of Materials ETH Zurich Zurich 8093 Switzerland

**Keywords:** amyloid polymorphism, atomic force microscopy, filament intertwining mechanism, mixed‐curvature amyloid

## Abstract

Amyloid polymorphism is a hallmark of almost all amyloid species, yet the mechanisms underlying the formation of amyloid polymorphs and their complex architectures remain elusive. Commonly, two main mesoscopic topologies are found in amyloid polymorphs characterized by non‐zero Gaussian and mean curvatures: twisted ribbons and helical fibrils, respectively. Here, a rich heterogeneity of configurations is demonstrated on insulin amyloid fibrils, where protofilament packing can occur, besides the common polymorphs, also in a combined mode forming mixed‐curvature polymorphs. Through AFM statistical analysis, an extended array of heterogeneous architectures that are rationalized by mesoscopic theoretical arguments are identified. Notably, an unusual fibrillization pathway is also unraveled toward mixed‐curvature polymorphs via the widespread recruitment and intertwining of protofilaments and protofibrils. The results present an original view of amyloid polymorphism and advance the fundamental understanding of the fibrillization mechanism from single protofilaments into mature amyloid fibrils.

## Graphical abstract

Polymorphism is a key feature of amyloid fibrils, yet the mechanisms remain elusive. This work presents a comprehensive AFM study and proves the hierarchical intertwining mechanism from single fibrils into mature amyloid fibrils, forming the mixed‐curvature amyloid polymorphs. The results advance the fundamental understanding of amyloid polymorphism and its fibrillization mechanism.

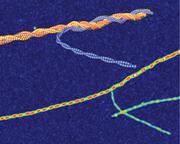

## 1 Introduction

Amyloid fibrils are highly‐ordered linear protein self‐assemblies formed via a general intermolecular cross‐*β* structure formation in the amyloid core.^[^ ^1^ ^]^ However, amyloids stemming from the same polypeptide chain often exhibit a rich morphological diversity across distinct amyloid species.^[^ ^2^ ^]^ This heterogeneity, also known as amyloid polymorphism, is a significant feature of amyloid fibrils that has been found in both pathological amyloids in vivo,^[^ ^3^ ^]^ and artificial amyloids in vitro.^[^ ^2^, ^4^ ^]^ For disease‐related amyloids, polymorphism was observed among Tau,^[^ ^5^ ^]^ Aβ peptide,^[^ ^6^ ^]^ *α*‐synuclein,^[^ ^7^ ^]^ and prion^[^ ^8^ ^]^ amyloids derived from both patients and animals, and its association with pathogenicity and diverse disease variants is suggested.^[^ ^3^ ^]^ In the context of artificial amyloids, polymorphism was extensively identified in numerous natural proteins such as *β*‐lactoglobulin,^[^ ^4^, ^13^ ^]^ lysozyme,^[^ ^2^, ^10^ ^]^ and oat‐globulin.^[^ ^11^ ^]^ The physical and morphological properties of amyloid polymorphs are exploited in applications as different as environmental remediation,^[^ ^12^ ^]^ materials science and biomedicine,^[^ ^13^ ^]^ liquid–liquid crystalline phase separation,^[^ ^14^ ^]^ and scaffolding.^[^ ^13^, ^15^ ^]^ It is therefore important to understand the structural origin and fibrillization mechanisms of these amyloid polymorphs. However, our current knowledge of the physical mechanism of amyloid polymorphism remains limited.

From a mesoscopic perspective, amyloid polymorphism arises as a consequence of distinct geometrical packings of a varying number of protofilaments^[^ ^2b^ ^]^ that constitute the mature fibril or distinct folding of the amyloid core.^[^ ^5c^ ^]^ Pathological fibrils including Tau and *α*‐Synuclein, and artificial amyloids are mostly composed of paired,^[^ ^5^, ^16^ ^]^ quadrupled,^[^ ^3^, ^17^ ^]^ or a larger, up to ten,^[^ ^2b^ ^]^ number of protofilaments. Traditionally, the arrangement of protofilaments into mature fibrils is based on two main packing schemes, namely twisted ribbons and helical ribbons,^[^ ^2^, ^18^ ^]^ characterized by saddle‐like and mean‐like curvatures, respectively.^[^ ^19^ ^]^ In general, the transition from twisted ribbon to helical ribbon occurs at a critical number of lateral packing filaments.^[^ ^2^, ^18^ ^]^ Alternatively, the protofilament‐packing can occur also in another mode, where two different topological curvatures can combine and form a new more complex structure that we name mixed‐curvature polymorph^[^ ^3^, ^4^, ^17^ ^]^ to underline its hybrid nature. Understanding the intricate protofilament packing within multistranded fibrils, coupled with their fibrillization process driven by the sequential recruitment of protofilaments,^[^ ^20^ ^]^ is key to elucidating the fundamental fibrillization mechanisms. Nevertheless, solving the structure of complex fibrils by techniques such as cryo‐Electron Microscopy (cryo‐EM) proves challenging due to the vast diversity of fibril polymorphs,^[^ ^4^ ^]^ and consequently these intricate mixed‐curvature fibrils and the mechanisms governing their fibrillization remain poorly understood.

In this study, we tackle the challenge posed by the extensive structural heterogeneity of insulin amyloid fibrils. By atomic force microscopy (AFM) statistical analysis, we categorized a broad spectrum of multistranded fibril polymorphs and further rationalized these fibril polymorphs using a concise set of rules for their hierarchical protofilament‐packing organization, characterized by their morphological fingerprints. Remarkably, our results substantiate that the packing of insulin protofilaments can bring about the formation of mixed‐curvature polymorphs. Our observations capture the evolution from the protofilaments into protofibrils and finally into mature fibrils and provide direct evidence of an unprecedented intertwining mechanism among protofilaments and protofibrils, to form higher‐ordered mixed‐curvature mature fibrils. This study advances our understanding of amyloid polymorphs, their formation, and evolution, and provides a solid ground for a refined classification of mesoscopic polymorphism.

## 2 Results and Discussion

**Figure 1:**
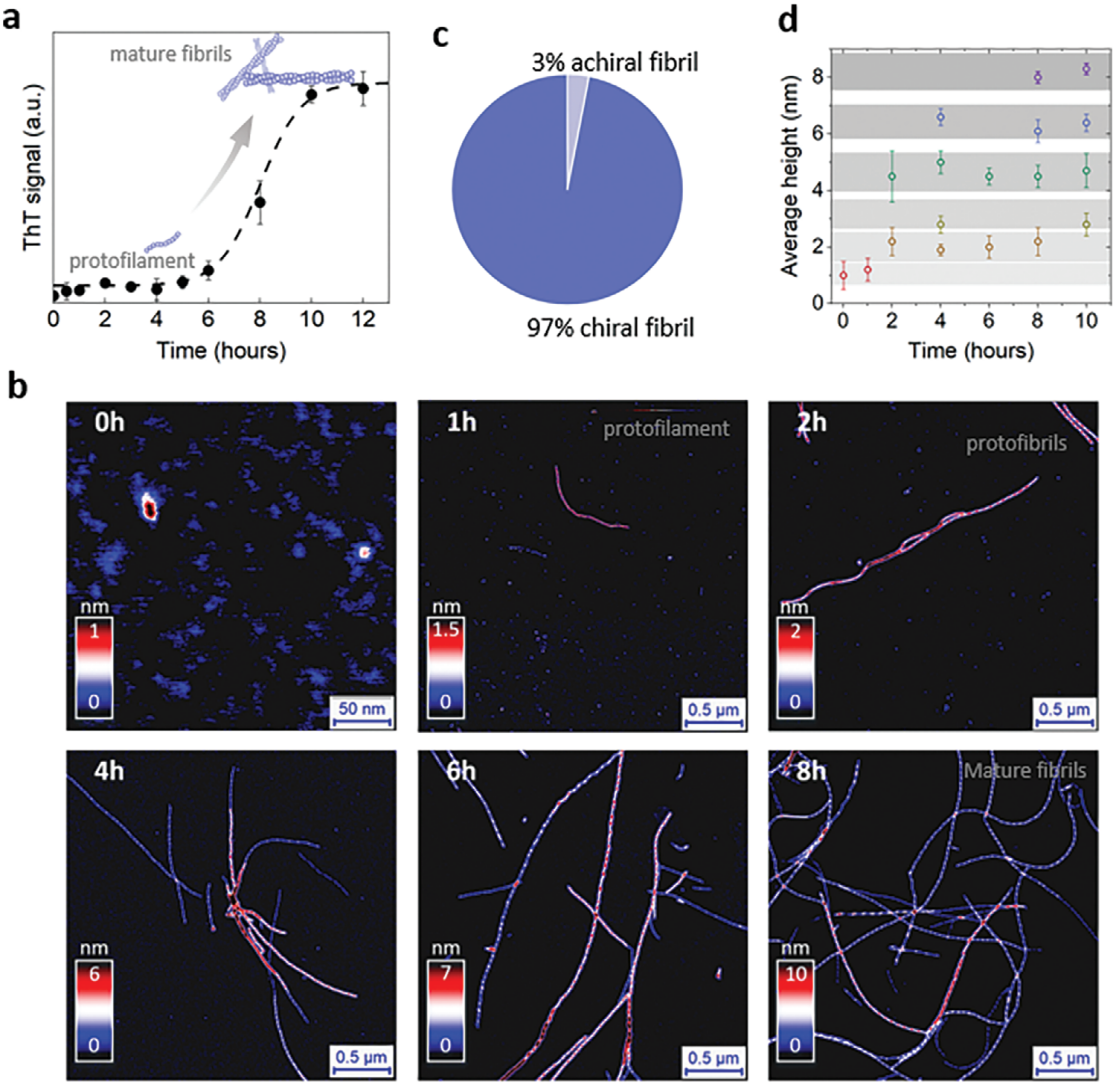
The evolution of insulin amyloid fibrils during incubation. a) Aggregation kinetics monitored by ThT fluorescence intensity as a function of time. b) AFM images of insulin aggregation, from monomers to protofilaments, protofibrils, and then mature fibrils at different time points (0, 1, 2, 4, 6, and 8 h) of incubation. Noticeable periodic fluctuations in fibril morphologies are evident. c) Relative abundance of fluctuating chiral fibrils within the whole insulin amyloid population. d) Evolution of average height of amyloid fibrils over incubation time showing the tendency for forming thicker fibrils with prolonged incubation.

In this study, we investigated insulin fibrillization and first traced the kinetics of insulin protein aggregation using Thioflavin T (ThT) fluorescence that showed a typical sigmoidal growth curve (**Figure** **1**a), in accordance with the nucleation‐dependent reaction model.^[^ ^4^, ^28^ ^]^ After a 4‐h lag phase for initial nucleation, an increasing *β*‐sheet structure emerged during the growth phase, leading to the final saturation phase. Circular dichroism (CD) and infrared (IR) spectroscopy also confirmed the clear transition from α‐helix to rich β‐sheet conformation during aggregation in the bulk protein solution (Figure S1, Supporting Information).

### 2.1 Morphological Evolution of Insulin Amyloid Fibrils

The morphological evolution of insulin fibrils during incubation was investigated by AFM as shown in Figure 1b and Figure S2 (Supporting Information). Following 1 h incubation, the initial monomeric protein was first assembled into single protofilaments with a diameter of 1 nm. In the following 2–3 h, a growing number of protofilaments and early protofibrils emerged, showing a diameter of 2–5 nm and a length of ≈2 µm. Subsequently, in addition to the early protofibrils, more and more thick and long mature fibrils, up to 9 nm in diameter, were observed, and finally, abundant mature fibrils were found in the saturation stage (Figure S2, Supporting Information). Interestingly, we found that most insulin protofibrils and mature fibrils, excluding single protofilaments, exhibited a left‐handed chirality with evident periodic height fluctuations, hence referred to as chiral fibrils (Figure 1b). We found that this chiral rule applies to most fibrils, irrespective of the fibril diameter and length, and the stage of fibrillization, accounting for 97% of the overall population (Figure 1c).

To gain insights into fibril evolution, we performed a statistical analysis on the average height of chiral fibrils across varying incubation periods. As shown in Figure S3 (Supporting Information), the average height distribution of fibrils steadily shifts toward higher values as incubation progresses, suggesting the emergence of thicker fibril architectures. Moreover, starting from ≈2 h incubation, this distribution becomes multimodal, pointing to the co‐existence of multiple fibril populations. Strikingly, the positions of peaks remain consistent throughout incubation and new peaks gradually emerge at greater height values (Figure 1d). This kinetics implies the presence of well‐defined fibril populations along the time course of incubation. The morphological evolution suggests a hierarchical fibril formation process, wherein it suggests the fibrillization mechanism of systematically packing protofilaments into multistranded fibrils.

**Figure 2:**
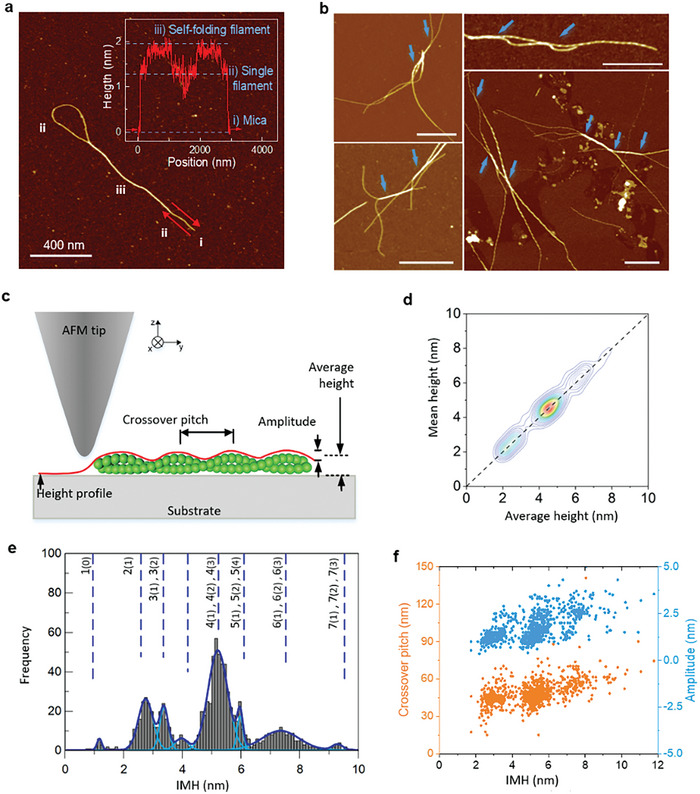
Fibril–fibril interaction and morphological investigation on insulin chiral fibrils. a) AFM snapshot of a self‐folded protofilament with a nanoracket‐type shape. The inset shows the height profile along the direction of the arrows. b) Snapshots of intertwining protofilaments and protofibrils at the early stage of fibrillization, indicated by the arrows. Scale bars are 500 nm. c) Schematic representation of a chiral fibril consisting of two protofilaments under AFM investigation. The extracted height profile (red) along the fibril ridge is used for calculating the average height, crossover pitch (crossover distance), and amplitude of the chiral fibril. d) Density map of the average height distribution computed from the full profile against the mean height calculated as the arithmetic mean between minimum and maximum height. e) IMH distribution of chiral fibrils obtained in the experiment, featuring seven distinct families peaked at 1.2 ± 0.2, 2.7 ± 0.4, 3.4±0.3, 5.1±0.7,6.0±0.3, 7.4±0.6 and 9.4±0.4 nm, corresponding to *n*  =  1,  2,  …,  7. An extra IMH peak located at 4 nm unveils the presence of a richer morphological complexity and possible packing arrangements are reported in Figure S8 (Supporting Information). f) Scatter plot of crossover pitch and amplitude against IMH of the chiral fibril. The clustered distribution in the plot indicates a correlation between the amplitude and the crossover pitch of chiral fibrils with their IMH height.

Such a hierarchical picture is further endorsed by direct evidence of the mutual intertwining of protofilaments and protofibrils across the various incubation stages (Figure S2, Supporting Information). This phenomenon is particularly prevalent during the initial lag and growth stages, in which traditionally the nucleation events (as illustrated in Figure S4, Supporting Information) are believed to play the main role.^[^ ^21^ ^]^ Interestingly, we also captured a self‐folding protofilament with a racket‐type shape (**Figure** **2**a), a geometry considered one of the initiating mesoscale building blocks of amyloid plaques.^[^ ^22^ ^]^ The protofilament has a height of 1.2 nm, closely resembling the height of insulin monomers, and upon self‐folding, its height jumps to ≈2 nm (Figure 2a). More AFM snapshots of early protofilament–protofilament intertwining are shown in Figure 2b, suggesting the widespread occurrence of braiding protofilaments into multistranded fibrils with complex configurations. We reason that this intertwining mechanism is rooted in the interplay between the mutual surface adhesion of protofilaments^[^ ^22^ ^]^ promoting attraction and their high surface charge responsible for long‐range repulsive interactions.^[^ ^9^ ^]^ We tested this hypothesis by growing fibrils at a high ionic strength condition and indeed observed bundles of amyloid fibrils, without apparent chiral morphology or hierarchical order (Figure S5, Supporting Information).

### 2.2 Statistical Analysis of Morphologically‐Fluctuating Chiral Fibrils

To deeper characterize the fibril–fibril intertwining and related amyloid architectures, we investigated the morphological fingerprints of the hierarchical chiral fibrils. Due to the tip‐sample convolution effect, AFM images do not perfectly describe the topology of multistranded fibrils (Figure S6, Supporting Information). However, this limitation can be circumvented by employing AFM high vertical resolution. We extracted the maxima from each cross‐section of the fibril, which is considered the most accurate data in the AFM image (Figure S6, Supporting Information), and then traced the maxima along the fibril ridge to obtain their height profiles, as illustrated in Figure 2c. Consequently, we acquired the fingerprints that depict the features of chiral fibrils: average height was obtained by averaging the height of all pixels along the fibril, as $H_{Av.}=\frac{1}{n}\sum_{k=1}^{n}H_{k}$, where *H_k_* refers to the height of each pixel on the fibril height profile; the crossover pitch (*P*) is the distance between consecutive peaks; the maximum (*H_max_*) and minimum (*H_min_*) height are computed as the values corresponding to peaks and wells on the height profiles respectively; the amplitude (*A*) is defined as *A*  = *H_max_*  − *H_min_*.

The histogram depicting the average height of chiral fibrils exhibited a multimodal hierarchy with six discernible peaks (Figure S7a, Supporting Information). This multimodal pattern aligns with the literature on other amyloid fibrils, where these peaks are usually ascribed to fibrils with varying quantities of protofilaments.^[^ ^9^, ^19^, ^23^ ^]^ However, we found that the average height is insufficient to discern various schemes because qualitatively‐different packing arrangements, may yield similar average heights, as illustrated in Figure S7c,d (Supporting Information). Alternatively, the maximal height can in principle discriminate better between these hierarchical fibrils,^[^ ^9^ ^]^ yet its distribution does not satisfactorily capture various multimodal peaks (Figure S7b, Supporting Information). To better characterize these polymorphic fibril populations, we introduce a novel metric, namely integrated maximal height (IMH), defined as:

(1) $$H_{IMH}=H_{Av}\;+\frac{1}{2}A$$

**Figure 3:**
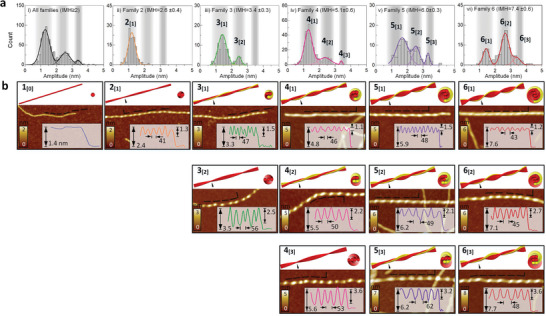
Classification of hierarchical configurations of chiral fibrils. a) Histogram of the amplitude distribution of all chiral fibrils with IMH height of more than 2 nm i) and chiral fibrils from IMH family 2–6 ii–vi). Regions shaded in grey refer to the IMH of fibrils with 1, 2, and 3 protofilaments. The labels on the peaks of the multimodal distributions refer to distinct fibril configurations. b) Schematic models of possible configurations of chiral fibrils consisting of up to 6 protofilaments with the cross‐section of each fibril model at the indicated position. The corresponding fibrils are illustrated in the AFM images and the insets show the height profiles of the respective fibril along the dashed lines, in which the IMH, crossover pitch, and amplitude are noted.

Before further characterization, we ensured the maxima and minima (and thus the IMH) as reliable indicators of the height profiles. To this aim, we compared the average height (*H_Av_*) with its estimation of mean height obtained as $H_{mean}=\frac{1}{2}\;\left(H_{max}+H_{min}\right)$ in Figure 2d. We found that these quantities are situated in the close neighborhood of the bisector of the first quadrant which confirms the consistency between the two estimates, supporting the robustness of IMH and amplitude in describing fibrils morphology. As expected, similar to the case of average height, IMH distribution also captures the presence of the multiple peaks (Figure 2e), located at 1.2 ± 0.2, 2.7 ± 0.4, 3.4 ± 0.3, 5.1 ± 0.6, 6.0 ± 0.3, 7.4 ± 0.6, and 9.4 ± 0.4 nm. Intriguingly, these values correspond to integer multiples *n* = 1, 2, …, 7 of the height of a single protofilament, based on which we accordingly designated these families as 1 to 7. These peaks are related to the maximum lateral extension of fibril packings, and hence they indicate the presence of different configurations. An extra IMH peak located at 4 nm unveils the presence of a richer morphological complexity and its possible packing arrangements in Figure S8 (Supporting Information) will be discussed later. We then investigated the association between IMH and other geometrical features. In Figure 2f, we present a scatter plot showing the variation of crossover pitch and amplitude as a function of IMH. The tendency of clustering data points implies the dependence of both crossover pitch and amplitude of chiral fibrils on their IMH height. Hence, we further analyzed these quantities within each family identified by the IMH distribution. For the crossover pitch, we found only one peak in the distribution of each family, exhibiting an overall linear increase as a function of IMH (Figure S9, Supporting Information), consistent with previous observations.^[^ ^9^ ^]^ Yet, a notable overlap of crossover pitch distributions was found among different families, introducing complexity to further characterization. Remarkably, for the amplitude, within each IMH family, we found instead distributions (**Figure** **3**a; Figure S10, Supporting Information) showing multiple regularly‐located peaks (1.3 ± 0.4, 2.5 ± 0.4, and 3.4 ± 0.2 nm). These values surprisingly correspond to the IMH of fibrils from families I, II, and III (1.2 ± 0.2, 2.7 ± 0.4, and 3.4 ± 0.3 nm, Figure 2e), suggesting that both IMH and amplitude can be rationalized by an integer number of protofilaments. Within this picture, the correlation between amplitude distribution peaks and the IMH values suggests that the different peaks of amplitude distribution within each family are related to distinct arrangements of protofilaments/protofibrils leading to the fibril family under inspection. These findings reinforce the hypothesis that chiral fibrils are hierarchically built by intertwining existing protofilaments or protofibrils.

### 2.3 Configurational Identification of Fibril Polymorphs

Based on the statistical analysis above, we propose a nomenclature for protofibril as *n*_[α]_ where IMH corresponds to *n*‐th family and the amplitude is symbolized by α protofilaments. For instance, notation 3_[1]_ denotes a fibril belonging to family 3 with an amplitude corresponding to 1 protofilament. We note that the *n*‐th family does not univocally correspond to the number of protofilaments *j* forming the fibril; rather, we posit that *j* ≥ *n*. By means of this definition, we conceptualized the possible packing arrangements of observed chiral fibrils according to their IMH and amplitude, as shown in Figure 3b. It is worth noting that our proposed insulin fibril configurations include as particular cases cryo‐EM‐solved structure in the literature,^[^ ^4^, ^24^ ^]^ which further supports our hypothesis.

Our classification includes two distinct categories of fibril polymorphs: twisted‐ribbon polymorphs and mixed‐curvature polymorphs. Twisted ribbons correspond to the cases in which *j*  =  *n* and α  =  *n* − 1, including 2_[1]_, 3_[2]_ and 4_[3]_ in Figure 3b, and are constructed by arranging protofilaments in a side‐by‐side stacking manner. This scheme was frequently reported,^[^ ^9^, ^10^, ^25^ ^]^ such as *β*‐lactoglobulin and lysozyme, and may arise from the recruitment of monomers or protofilaments through specific interactions such as steric zipper.^[^ ^24^, ^26^ ^]^ We noticed that early twisted ribbons (3_[2]_ and 4_[3]_) become less and less abundant (Figure 3a (iii,iv)). On the other hand, mixed‐curvature polymorphs correspond to *n* ≥ 3 and α < *n* − 1 (Figure 3b) and are likely composed of two intertwining protofilaments or early protofibrils. In these cases, the condition *j*  =  *n* does not necessarily hold (see Figure S8b for an example of class *n*  =  4, *j*  =  5, Supporting Information). Such polymorphs are mostly found in pathological amyloids in vivo, including serum amyloid‐A^[^ ^3b^ ^]^ and Drosophila Orb2 protein,^[^ ^17a^ ^]^ in which their fibrils consisting of three or four protofilaments can be classified according to our rationale. We believe this scheme may arise from protofibril surface adhesion which is the combination of interactions including hydrophobicity and electrostatic interaction.^[^ ^9^, ^22^, ^27^ ^]^

Besides, our statistical data also indicate a non‐monotonic behavior of fibrillization propensities. For instance, fibrils of class *n*  =  4 are the most common configuration among all populations (Figures 2e and 3a). Thin fibrils (*n* ≤ 4) tend to progressively combine with single protofilaments and therefore fibrils 3_[1]_ and 4_[1]_ dominate each family (Figure 3a). In contrast, thick fibrils (*n* ≥ 5) are mostly formed by recruiting protofilament pairs (*n*  =  2) due to the majority of fibrils 5_[2]_ and 6_[2]_ in each family. We ascribe this feature to the observation of the progressive disappearance of single protofilaments from the system (Figure S3, Supporting Information). At the early stages of aggregation, thin fibrils are then formed by progressively attaching single protofilaments, whose presence is still presumably abundant, which explains the dominance of 3_[1]_ and 4_[1]_ for early fibrils (*n* ≤ 4). It is plausible that at a critical thickness (e.g., *n*  =  4 or larger) the single protofilaments are already close to being exhausted and thus, recruiting pairs become statistically more favorable. We further note that protofilament depletion may also occur simultaneously with a change in the hydrophobic/electrostatic balance evolving along with polymorphism, which can further play a role in the final observed populations of polymorphs.

From a mesoscopic perspective, the two classes of polymorphs can also be separated by considering the mean (*H*) and Gaussian (*K*) curvatures of amyloid fibril surfaces. For twisted‐ribbon polymorphs, mesoscopic bending of protofilaments in the twisted ribbon is minimal, while *K* deviates from zero due to this saddle‐like topology^[^ ^2b^ ^]^ and thus H ≈ 0 and K ≈ −1/[L/2π]^2^, where periodicity (L) of fibrils refers to twice the crossover pitch (L  =  2P).^[^ ^16b^ ^]^

In general, the saddle‐like twisted ribbon could transit into the helical ribbon that exhibits non‐zero mean curvature and zero Gaussian curvature (*H* ≈ −1/2*R*; *K* ≈ 0, where *R* is the radius of the helical ribbon) at a critical number of lateral packing protofilaments.^[^ ^2^, ^18^ ^]^ Yet, helical ribbons were not observed during the insulin fibrillization process since the twisted‐ribbon structures did not reach the critical number of protofilaments for helical fibril formation. In contrast, an alternative pathway to reduce the overall elastic and chiral attraction energy is to form mixed‐curvature polymorphs, where both torsion and bending contributions are present. Experimentally, we observed that the mixed‐curvature polymorphs are more prevalent than twisted ribbon polymorphs, particularly at longer incubation times, suggesting that these mixed‐curvature structures possess a lower associated energy.

### 2.4 Cryo‐EM Classification Verifies Twisted‐Ribbon and Mixed‐Curvature Polymorphs

**Figure 4:**
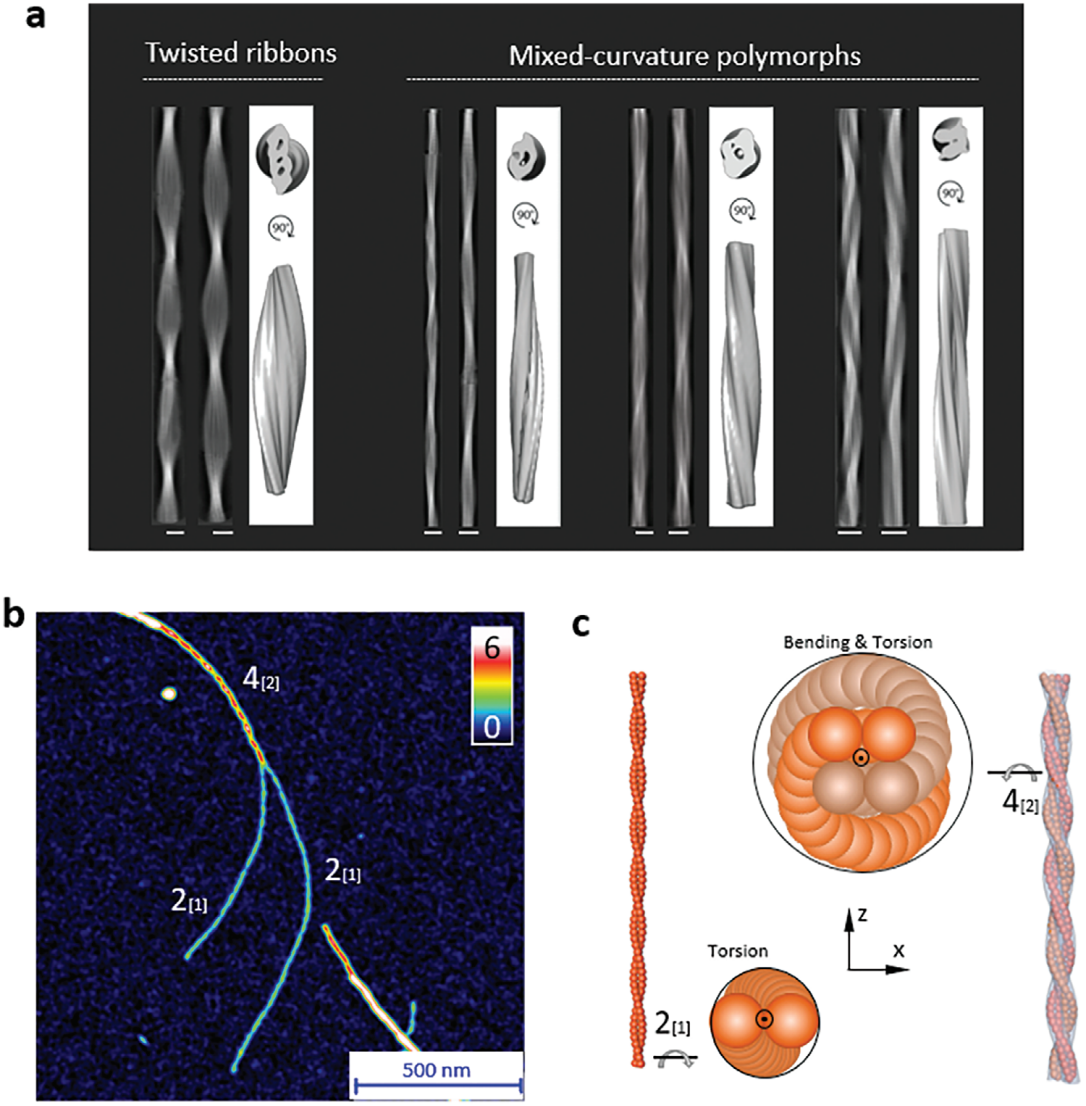
Cryo‐EM observation of fibril polymorphs and the intertwining of early protofibrils. a) The reconstituted fibrils (stitched 2D class averages) on the left side of the panels illustrate the heterogeneous nature of individual fibrils for each polymorph, which includes a twisted ribbon and three mixed‐curvature polymorphs. In each case, a 3D model of each polymorph displays the 3D averaged cross‐sections and side views, further highlighting the differences between polymorphs. Scale bars represent 8 nm. b) A snapshot of the intertwining of two identical early protofibrils 2_[1]_ into the higher‐ordered protofibril 4_[2]_. These protofibrils are identified by their height fingerprints in Figure S11 (Supporting Information). c) The protofilament‐based schemes of the protofibrils from panel b, as a representative model demonstrating the transition from twisted ribbons into mixed‐curvature polymorphs. Twisted ribbon 2_[1]_ involves the torsion of protofilaments, while the merged 4_[2]_ protofibril, formed by the helical intertwining of two twisted ribbons, has non‐zero torsional and bending energy.

The identification of our proposed two categories of fibril polymorphs was further validated by cryo‐EM. We explored the dominating chiral fibrils (Figure 1c) and found four fibril morphologies, that are one twisted‐ribbon polymorph and three mixed‐curvature polymorphs, as shown in the 2D class average images and corresponding initial 3D models in **Figure** **4**a and Figures S11–S13 (Supporting Information), These polymorphs are composed of highly‐ordered, twisting *β*‐sheet protofibrils, which are aligned in a side‐by‐side stacking or in a more complex stacking geometry to form a thicker fibril construct (Figures S11–S13, Supporting Information). The herein rather high structural heterogeneity and variability of the crossover distances prevented a higher‐resolution 3D analysis of the fibrils (Figure 4a; Figure S11, Supporting Information).

In line with AFM observation, we confirmed that the mixed‐curvature fibril in Figure S11c,d (Supporting Information) is the most abundant polymorph among the reconstituted polymorphs after 8 h incubation. Besides, we found two types of thin mixed‐curvature protofibrils: the first type (Thin1, Figure S11a,b, Supporting Information) displays large individual differences; the second (Thin2, Figure S11e,f, Figure S13 red classes, Supporting Information) with fewer particles was identified upon a closer examination. These thin protofibrils are more prevalent at an earlier time point (4 h), while the thick and thin filaments are equally abundant at later stages. This suggests their role as building blocks for forming thicker higher‐ordered polymorphs. In contrast, we found that twisted ribbons (Figure S11g,h, Supporting Information) are present throughout the fibrillization process and constitute a smaller portion of the polymorphs.

### 2.5 Direct Observations of Intertwining Protofibrils into Mixed‐Curvature Fibrils

Our proposed hypothesis of hierarchical amyloid fibrillization and mixed‐curvature polymorphs formation is corroborated by direct observations. In Figure 4b, we illustrate an interesting event where two early protofibrils intertwine. By evaluating their AFM profiles (Figure S14a, Supporting Information) in light of our statistical data (Figure 3a), we identified this event as the merging of two 2_[1]_ protofibrils forming a higher‐order 4_[2]_ fibril. Interestingly, this observation serves as a representative model demonstrating the transition from twisted ribbons to mixed‐curvature polymorphs. Specifically, the individual 2_[1]_ protofibrils are twisted ribbons with inherent torsional energy, while the merged 4_[2]_ protofibril is composed of two protofibrils 2[1], winding along a helical repeat (Figure 4c; Figure S15, Supporting Information), while maintaining the inherent torsion in each protofibril. Therefore, both torsional and bending energy are involved in the formation of mixed‐curvature polymorphs. In nature, such mixed structures can often be found including the wrapped DNA around histones and protein coiled coils. Another intertwining case is shown in Figure S14b (Supporting Information), which demonstrates the 4_[1]_ and 2_[1]_ protofibril intertwining to form a 6_[2]_ fibril. These observations further reinforce our hypothesis that higher‐ordered insulin mixed‐curvature fibrils form through the intertwining mechanism of early fibrils including protofibrils and protofilaments.

**Figure 5:**
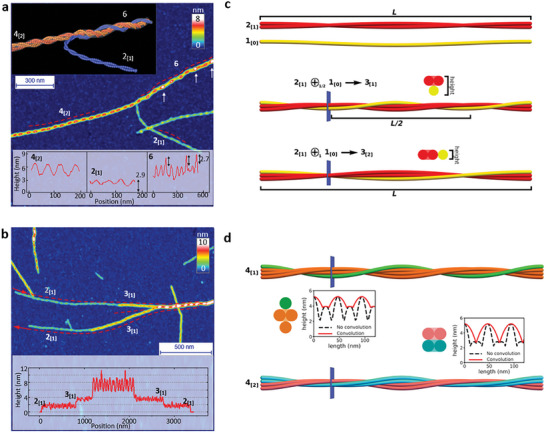
Visualization of protofibril intertwining and the consensus model for fibril formation. a, b) AFM snapshots of the a) intertwining of 2_[1]_and 4_[2]_ protofibrils into higher‐ordered fibrils in family 6 and b) of the entangling between two identical 3_[2]_ protofibrils that are both formed by 2_[1]_ protofibrils, recruiting another protofilament and generating a higher‐ordered family‐7 fibril. The inset shows the AFM‐profile fingerprint of chiral fibrils before and after intertwining along the dashed lines and arrows in the images. c) Representative case of the combination of protofibrils into mature fibrils, showing a protofibril 2_[1]_ and a protofilament 1_[0]_ combine into a three‐stranded fibril, either with *k*  =  1 (2_[1]_⊕_1/2_1_[0]_ →  3_[1]_) or with *k*  =  2 (2_[1]_⊕_1_1_[0]_ →  3_[2]_). The right sections corresponding to the blue plane are depicted, and the corresponding height is indicated. d) Structures and AFM profiles for the four‐stranded fibrils 4_[1]_ (left) and 4_[2]_ (right). In the AFM profiles, the real height is depicted as a black dashed line, while red continuous lines give the height obtained after convolution with a tip with an effective radius *R*  =  90 nm.

Furthermore, we report two more complex cases of this intertwining fibrillization. In **Figure** **5**a, we report an intriguing scenario where distinct hierarchical height profiles can be discerned. The composing protofibrils are identified as 2_[1]_ and 4_[2]_ according to their height profiles. Upon intertwining, they form a thicker fibril belonging to family 6. In contrast with the cases reported in Figure 3b, the AFM profile of the mature fibril shows two sets of peaks, one corresponding to the typical IMH of family 4 and the other to family 6. In Figure 5b, two identical protofibrils 3_[1]_, both formed by braiding of a protofibril 2_[1]_ and a protofilament (denoted as 1_[0]_), intertwine into a higher‐order fibril. The IMH of the final fibril corresponds to that of family 7, which leads us to speculate that it is the result of the combination of the two *n*  =  3 protofibrils into a family 6 fibril, which then recruited another protofilament while intertwining, resulting in a thicker 7_[3]_ fibril.

### 2.6 Morphological Modeling of Chiral Fibrils

Starting from the results of the statistical analysis, we built a consensus model for the formation of hierarchical fibrils. As discussed above, we assume no interstitial space to be present between adjacent protofilaments, which imposes that a fibril belonging to the family *n* is formed by at least *n* protofilaments. Unfortunately, the information provided by IMH and amplitude cannot unambiguously indicate the exact composition of the fibrils, hence we proceeded by first building packing arrangements corresponding to the simplest scenario, where exactly *n* protofilaments are assigned to a family *n* fibril. Strikingly, this simplistic approach is capable of recapitulating most (although not all) experimental observations.

In order for a fibril with *n* protofilaments to reach the IMH corresponding to family *n*, somewhere along the fibril all the protofilaments must be aligned vertically. Within this framework, combining two protofibrils *n*_[α]_ and *m*_[β]_ while maintaining the existence of this vertical alignment yields an IMH corresponding to *n* + *m* protofilaments. Microscopically, this implies that the adhesion patches of the protofilaments must have a suitable orientation with respect to each other. The amplitude instead depends on the specific way in which the protofibrils intertwine. To reproduce the coherency observed in the experiments, we assume that the protofibrils intertwine with the period *k*/2 · *L*, where *k* is a positive integer and *L*  =  94 nm is a reference period (Figure S9a, Supporting Information), and denote this combination by using the symbol ⊕_*k*/2_. For instance, in Figure 5c we show the combination of 2_[1]_ and 1_[0]_ into three‐stranded fibrils: choosing *k*  =  1, we obtain 2_[1]_⊕_1/2_1_[0]_ →  3_[1]_, where the two‐stranded protofibril is twisting with period *L*, while the protofilament is twisting twice as fast, corresponding to the case in Figure 5b; for *k*  =  2, we instead obtain 2_[1]_⊕_1_1_[0]_ →  3_[2]_.

The assembly operator ⊕_*k*/2_ allows building periodic structures with complex height profiles (dashed lines in Figure 5d). We speculate that the single periodicities detected in the majority of experimental observations are due to the convolution of the AFM tip on such complex profiles. To estimate the effect of the AFM tip due to its shape and the operating mode, we consider a simple model in which the tip is a hard sphere with an effective radius *R* and the measured height is obtained as the lowest point at which no overlap occurs between the tip and the filaments (Figure S16, Supporting Information). In Figure 5d (red lines), we report the convoluted profiles obtained for *R*  =  90 nm, which shows that AFM tip convolution can indeed introduce single‐period profiles starting from more complex morphologies. The value of *R* is large when compared to the apex radius of the probes used in this work (≈7 nm); in this regard, we note however that the measurements were taken in noncontact mode, hence it is expected that convolution effects correspond to a larger effective radius. The quantitative accuracy of the estimation considered here, *R*  =  90 nm, can be gauged by observing that this value corresponds to an apparent protofilament width equal to $w\;=\;2\sqrt{2Rb}≃30$ nm (see Estimation of the Apparent Width of Protofilaments in Section 4), in line with the experimental observations (Figure S6, Supporting Information).

Based on our model, we found that *k*  =  1 and *k*  =  2 cover most of the observed profiles. As mentioned above, we ascribe the single periodicity observed in the experiments to the convolution of the AFM tip on the complex height profile of the fibril (Figure 5d). For the chosen values of *k*, one thus effectively detects a minimum in the correspondence of the secondary maximum, which is located at a distance *L*/4 from the vertically aligned configuration (Figure 5d). The only exception is provided by the twisted ribbon, which is already single‐peaked in the original profile; in this case, the position of the detected minimum corresponds with the absolute “true” minimum (Figure S17, Supporting Information), which is again located at a distance *L*/4 from the vertically aligned configuration. Note also that different choices of *k* > 2 predict a complex profile also after convolution, as discussed below (Figure 5a; Figure S18, Supporting Information).

In the case of *k*  =  1, the detected minimum is obtained by setting the minimum configurations of the protofibrils on top of each other (Figure 5c). The minimum configurations of protofibrils *n*_[α]_ and *m*_[β]_ have heights equal to (*n* − α) · *b* and (*m* − β) · *b*, hence the combined profile has a minimum height equal to (*n* + *m* − α − β) · *b*. Since the maximum height is (*n* + *m*) · *b*, we thus conclude that the newly knitted fibril possesses an amplitude of (α + β) · *b*, i.e., we obtain the combination rule *n*_[α]_⊕_1/2_ *m*_[β]_ →  (*n* + *m*)_[α + β]_. Particularly, this allows for the formation of fibrils (*n* + 1)_[1]_ by combination of *n*_[1]_ and a single protofilament (e.g., 2_[1]_⊕_1/2_1_[0]_ →  3_[1]_ as in Figure 5b,c); moreover, it correctly explains the observed combination of two 2_[1]_ protofibrils into a 4_[2]_ fibril as 2_[1]_⊕_1/2_2_[1]_ →  4_[2]_ (Figures 4b and 5d).

For *k*  =  2, at position *L*/4 the protofibrils are again in their minimum‐height configurations, but due to the combination rule they are now lying next to each other (e.g., 2_[1]_⊕_1_1_[0]_ →  3_[2]_ in Figure 5c). The combined height at the minimum is thus obtained as γ · *b*, where γ  =  max(*n* − α,  *m* − β); the combination rule reads *n*_[α]_⊕_1_ *m*_[β]_ →  (*n* + *m*)_[*n* + *m* − γ]_. This rule accounts for the formation of twisted ribbons by incremental addition of a single protofilament, e.g. 1_[0]_⊕_1_1_[0]_ →  2_[1]_ and 2_[1]_⊕_1_1_[0]_ →  3_[2]_.

Despite the simplistic approximations, the proposed combination rules with *k*  =  1,  2 recapitulate all the combinations of IMH and amplitude reported in Figure 3, together with the direct observation of protofibrils intertwining in Figure 4, which corresponds to 2_[1]_⊕_1/2_2_[1]_ →  4_[2]_. Nevertheless, as mentioned above this model is by no means the only possible arrangement explaining the data. A clear indication of the higher complexity of morphologies present in the system is provided by the small IMH peak located at ≈4 nm in Figure 2e, which is located halfway between *n*  =  3 and *n*  =  4. This is clearly incompatible with a construction in which all the protofilaments are eventually aligned vertically. As shown in Figure S8 (Supporting Information), relaxing this assumption (which implies that a fibril of family *n* can have more than *n* protofilaments) easily leads to proposed structures that are compatible with this peak, obtained as the intertwining of twisted ribbons of two and three protofilaments. Another example of an outlier comes from the direct observation of Figure 5a, where the complex height profile of the obtained six‐stranded fibril can be obtained by combining the composing protofibrils via a combination rule with *k*  =  3 (Figure S18, Supporting Information). All in all, this suggests that this construction, on its full generality, can potentially account for the experimental observations, paving the way for more accurate descriptions if further empirical information becomes available in the future.

## 3 Conclusion

In summary, we performed a comprehensive study on insulin amyloid polymorphs using AFM statistical analysis, and we identified the hierarchical protofilament‐packing configurations within these heterogeneous chiral fibrils. We categorized these fibrils into two distinct polymorphs, twisted‐ribbon polymorphs and mixed‐curvature polymorphs, which significantly extends the palette of possible arrangements of amyloid fibrils. There are not only amyloid fibrils characterized simply by either mean or Gaussian curvature, but also mixed‐mode conformations featuring both bending and torsion. Furthermore, we demonstrated that the process of mature fibril formation is driven by the gradual and hierarchical intertwining of early fibrils including protofibrils and protofilaments. This is supported by AFM visualization and by our theoretical construction. Besides, our data also provide insights into the fibrillization tendencies such as the preferable protofilament packing configuration and the population of each variant.

Our results provide a valuable insight into the diverse architectures of amyloid polymorphs and unveil a fibrillization pathway of amyloid polymorphs through the recruitment or intertwining of protofilaments and protofibrils. This work sheds light on the mesoscopic mechanisms of amyloid formation and advances our understanding of the role of inter‐protofilament interaction in the formation of both functional and pathological amyloid fibrils.

## 4 Experimental and Theoretical Section

### Preparation of Insulin Fibrils

Bovine insulin (Sigma Aldrich, I5500) was obtained from the bovine pancreas, and other chemicals were also purchased from Sigma–Aldrich. Insulin powder was dissolved in the acidic salt‐free buffer (pH 1.6, 25 mm HCl) at a concentration of 1.5 mm. Immediately after that, the protein solution was vortexed for 1 min followed by a sonication for 5 min, and then adjusted to pH 1.6 before storing at 4 °C for 1 h for further dissolving. The solution was purified with a 0.22 µm filter and then diluted to the concentration of 1 mm followed by re‐adjusting to pH 1.6. The insulin solution was incubated at 80 °C. An aliquot solution (30 µL) was collected upon incubation time and immediately diluted ten times before storing at 4 °C to minimize further protein assembling for further analysis.

### Atomic Force Microscope (AFM) Measurements

An aliquot (10 µL) of fresh insulin self‐assembly solution (10 µm) was deposited on freshly cleaved mica for 2 min, followed by a gentle rinsing of 500 µL Milli‐Q water and then dried by a gentle flow of nitrogen gas under ambient conditions. The AFM samples were further dried and stored in a vacuum desiccator. AFM images were scanned by NX‐10 Atomic Force Microscopy (Park Systems, South Korea) using the noncontact Amplitude Modulation (NC‐AM) in ambient conditions. AFM images were scanned by using non‐contact cantilevers (PPP‐NCHR, Park) with a nominal resonance frequency of 330 kHz and nominal force constant of 42 N m^−1^ at a resolution of 1024 × 1204 pixels. Noting that appropriate controlling of the cantilever plays a key role in the statistical analysis of each AFM height measurement, a gentle tip‐sample interaction was carefully achieved by monitoring AFM phase images in the negative range with a phase shift less than ±5°. AFM images were flattened by XEI software (Park System, South Korea). To avoid artifacts induced in flattening and to compare data between different measurements, every AFM image was flattened by plane in the first order. The reliability of AFM scanning was monitored by controlling the roughness of the mica surface to less than 0.2 nm during imaging.

### Statistical Analysis of the Amyloid Fibril Morphologies

The flattened AFM images were analyzed with DNA trace software.^[^ ^28^ ^]^ This allowed to trace the height profile of the ridge of amyloid fibrils along their contour length, by either extracting the maximum point or using Gaussian fit in each cross‐section of the amyloid fibril. To homogenize the extracted data, a constant sampling step of 1 nm was used in each extraction of height profiles. To avoid artifacts, the fibrils with a minimal length of 150 nm were only taken into account and fibrils overlapped with each other were excluded. The features including the height value and position of peaks and dips on the fluctuating height profile were extracted, to calculate the morphological fingerprints of amyloid fibrils such as the average height, maximal and minimal height, amplitude, and crossover pitch.

### CD Spectroscopy

An aliquot (70 µL) of insulin solution at the initiating monomeric concentration of 30 µm was analyzed at room temperature with a Jasco J‐815 CD spectrometer. A high‐quality quartz cuvette with an optical path length of 1.0 mm was used and spectra were collected with a step 0.2 nm in a continuous scanning mode in the range of 190–280 nm in each measurement. The spectra were smoothed with a Savitzky–Solay filter in Origin.

### ThT Assay

An aliquot of insulin solution was diluted by freshly prepared ThT solution. In order to perform the ThT assay, a dilution factor of 500 times to reach a final protein concentration of 3 µm was carried out. An aliquot (70 µL) of diluted insulin solution with a ThT concentration of 10 µm in each experiment was measured three times in a Bucher Analyst AD plate reader and ThT fluorescence reading was performed at an excitation wavelength of 450 nm and an emission wavelength of 485 nm. The signal of fluorescence intensity was fitted with a sigmoidal model.

### IR Spectroscopy

An aliquot (5 µL) of diluted insulin solution (20 µL) was deposited on a hydrophobic ZnSe monocrystal prism at room temperature and was dried by evaporation in a vacuum desiccator overnight to form a thin layer of insulin aggregates on ZnSe surface. The freshly prepared sample was measured by the nanoIR system (Anasys Instruments Inc., USA) that combined AFM and infrared spectroscopy measurements^[^ ^29^ ^]^ and enabled chemical analysis at the nanoscale. A soft cantilever (EX‐C450, Anasys) with a spring constant of 0.2 N m^−1^ was used and the spectra were collected with a sampling rate of 1 cm^−1^ and 128 co‐average in the range of 1200–1800 cm^−1^, and further nominalization was realized in Analysis Studio (Analysis). The analysis of secondary structural compositions in the amide I (1600–1700 cm^−1^) was performed in Origin according to the correlations between protein structures and Amide I frequency indicated before.^[^ ^30^ ^]^ The spectra were processed according to previous methods,^[^ ^29a^ ^]^ and sub‐bands in the amide I region (1600–1700 cm^−1^) were revealed by multiple Gaussian fitting,^[^ ^30^ ^]^ including helical and turn‐like (1648–1685 cm^−1^) structure, *β*‐sheet and antiparallel *β*‐sheet (1610–1640 and 1685–1695 cm^−1^) structure and random coil (1640–1650 cm^−1^) structure.

### Cryo‐EM of Insulin Fibrils

Assembled insulin fibrils at a concentration of 30 µm in Milli‐Q water (pH 1.6) were adsorbed to glow‐discharged holey carbon film grids, which were plunge frozen with 5–6 s blotting time using Leica EM GP2 (Leica Microsystems AG, Switzerland). Grids were transferred into a Thermo Fisher Scientific (TFS) Titan Krios G4 electron microscope, equipped with a cold‐FEG electron source operating at 300 kV, SelectrisX energy filter (10 eV zero loss window), and dose‐fractionated “movies” were recorded as electron event recordings (EER) on a Falcon 4i camera. Data were collected at a total dose of 50 e Å^−2^, a pixel size of 0.658 Å, and a defocus range of 0.8–2.4 µm. Micrographs were analyzed during recording with cryoSparc Live.^[^ ^31^ ^]^ On‐the‐fly aligned micrographs were imported to RELION‐4.0.^[^ ^32^ ^]^ The contrast transfer function (CTF) was estimated with CTFFIND4.1.^[^ ^33^ ^]^ Fibrils were manually picked from the micrographs. A box size of 960 pixels was used first to extract all the picked particles. Reference‐free 2D classification was performed on the extracted particles, and different types of fibrils were then separated according to the rough measurement of the crossover distances, appearance, and width of the fibrils. Afterward, different fibril types were grouped and extracted separately using a box of 360 pixels, and 2D classification was performed on the extracted particles for several rounds to remove the wrong fibril types and images containing ice. Well‐resolved 2D classes were mapped back to their original positions by using the computational reconstitution approach described earlier.^[^ ^34^ ^]^ The reconstituted fibrils provided detailed information on four different filament types, here termed thin1, thin2, intermediate, and thick insulin fibrils. The mean crossover distance of each filament type was measured and 3D models were built by the Inimodel2D function of RELION‐4.0. Three types of filaments were present both in 4 and 8‐h assemblies. In 4‐h assembly, thin1‐2 (69%) fibrils were vastly more abundant than intermediate (13%), and thick (17%) while 8‐h assembly had less thin1‐2 (28%), more intermediate (16%), and thick (30%) fibrils. The crossover distances (100 measurements) and widths (50 measurements) for each of the four polymorphs were manually measured using Fiji.^[^ ^35^ ^]^ The measurements were then transferred to Microsoft Excel, where the mean and standard deviation were calculated.

### Theoretical Modeling of Insulin Fibrils and Construction of Fibrils

Models of the fibrils were built hierarchically starting from smaller components. An overall periodicity *L*  =  94 nm was fixed and the fibrils were built within an interval spanning several periodicities. A reference frame was considered in which the x axis is oriented along the fibril axis, the z is perpendicular to the substrate and the y axis is such that $\hat{y}=\hat{z}\;\times \hat{x}$, where the hat indicates the unit vector parallel to a given axis. The fibril models were built ensuring that for each section the combining protofibrils are in contact and oriented according to the prescribed phase, as detailed below. For each period, 800 equally spaced sections along the fibril axis were considered.

The details for building a generic fibril according to the rule $n_{\left[\alpha \right]}{⊕}_{\frac{k}{2}}m_{\left[\beta \right]}$ are as follows. For a given point x along the fibril axis, the sections of fibrils $n_{\left[\alpha \right]}$ and $m_{\left[\beta \right]}$ (oriented according to their own prebuilt configurations) were combined along the phase ϕ  =  2π*x*/(*k*/2), defined as the angle between the line joining the centers of the protofibrils and the $\hat{y}$ axis. The distance between the centers was fixed in order to ensure one contact point between the protofibrils while avoiding overlapping. In practice, this was obtained numerically by applying the bisection method. In the case of complex protofibrils (i.e., *n* > 1 or *m* > 1), the contact was obtained between each protofilament of one protofibril and the stadia corresponding to internal contact points of the other protofibril, in order to avoid artificial complex geometrical patterns coming from having chosen circular sections of the protofilaments.

As a practical example, consider the process $2_{\left[1\right]}{⊕}_{\frac{1}{2}}1_{\left[0\right]}\to \;3_{\left[1\right]}$, for which the final structure and a representative section are depicted in Figure S15a (Supporting Information). As shown in the right panel of the figure, the phase ϕ is defined as the angle formed by the $\hat{y}$ axis (horizontal line) and the line connecting the centers of 2_[1]_ and 1_[0]_. Indicating the two centers as *C*_1_ and *C*_2_, this means that *C*_1_ = *C*_2_  + *t*(cos ϕ, sin ϕ) for a certain *t*. The parameter *t* is determined by bisection. Starting from two values *t*_↓_ =  0 and *t*_↑_ = (*n* + *m* + 1) *b*  =  4*b* (chosen to be certainly too small and too large, respectively), the optimal *t* is found iteratively: at each iteration, by defining *t_try_* = (*t*_↓_  + *t*_↑_)/2, one checks whether *C*_1_ = *C*_2_  + *t_try_* (cos ϕ,sin ϕ) results in an overlap of the sections or not. In the former case, one assigns *t*_↓_ = *t_try_* , otherwise *t*_↑_ = *t_try_* . The procedure is repeated until *t*_↑_ − *t*_↓_ < 10^−4^ nm. As mentioned above, to avoid spurious effects due to the chosen circular shapes, the overlap is checked between the protofilament and the whole stadium formed by the components of 2_[1]_ (shaded red region in the section in Figure S14a, Supporting Information). With these rules, one can combine fibrils of increasing complexity.

### Computation of Theoretical AFM Profiles

The AFM height profiles were computed assuming that, at each section, the fibrils have a contact point with the substrate. Consider a fibril $n_{\left[\alpha \right]}$ and a section located at a certain x . The assumed existence of a contact point allows the direct computation of the height *h_noconv_* (*x*) in the absence of convolution: denoting as *z_i_* (*x*) the *z* value of the center of protofilament *i* (*i*  =  1, …, *n*) within the section, one has simply *h_noconv_* (*x*) = max {*z_i_* (*x*), 1 ≤ *i* ≤ *n*}  − min {*z_i_* (*x*), 1 ≤ *i* ≤ *n*} + *b*, where *b* is added to account for the finite size of the protofilament.

To account for the convolution with the AFM tip (here assumed to be a sphere of radius *R*), define the position of the tip I as $\Gamma (x)=(x,0,z_{\mathrm{TIP}})$. Then, for each section $x^{\prime }$, the value $z_{\mathrm{TIP}}(x,x^{\prime })$ resulting in contact between the tip and the set of spheres of diameter *b* centered at the points (*x*′, *y_i_* (*x*′), *z_i_* (*x*′)), with *i*  =  1, …, *n* is computed. This is similar in spirit to the approach followed above to build the fibrils, although here the overlap is computed in the three dimensions. It is noted that the computation is restricted to those sections for which contact with the tip is possible. Furthermore, in analogy with the computations above, for intra‐fibril contacts, the overlap between the tip and the spherocylinder corresponding to the protofilaments involved in the contact is considered. From this computation, $z_{\mathrm{TIP}}(x,x^{\prime })$ is then obtained. Then, the full 3D profile of the fibril is accounted for by computing ${\bar{z}}_{TIP}\;\left(x\right)=\max_{x^{\prime }}\;\left\{z_{TIP}\left(x,x^{\prime }\right)\right\}$. Finally, the convoluted height is obtained as $h_{conv}\;\left(x\right)={\bar{z}}_{TIP}\;\left(x\right)-R$.

### Estimation of the Apparent Width of Protofilaments

Consider a virtual AFM measurement in which a single protofilament was being scanned in the direction perpendicular to its axis. Within the present model, its cross‐section is a circle of radius *b*/2. When the AFM tip is far away from the protofilament, it touches the substrate and has a height equal to 0. In contrast, when it is found in the vicinity of the protofilament, it stops before touching the substrate because it makes contact with the protofilament. The transition between these two cases is represented in Figure S16b (Supporting Information), where the AFM tip is found in a position that enables touching both the substrate and the protofilament. The apparent width *w* of the protofilament corresponds to twice the horizontal separation between the centers of the tip and of the section of the protofilament and satisfies the relation $w\;=\;2(R+\frac{b}{2})\cos\theta$. Here, *θ* is the angle formed by the line joining the two centers and the horizontal. It is evident from Figure S15b (Supporting Information) that $\sin\theta =\frac{R-b/2}{R+b/2}\;$, thus $\cos\theta =\sqrt{1-\sin^{2}\theta }\;=\frac{\sqrt{2Rb}}{R+b/2}\;$, finally yielding $w\;=\;2\sqrt{2Rb}$.

## Conflict of Interest

The authors declare no conflict of interest.

## Author Contributions

J.Z., G.D., F.S.R., S.K.S., and R.M. conceived and designed the study, J.Z., M.T., and J.T. performed the experiment and data analysis, S.A. I.M.I., E.L.S., A.C., and R.M. contributed to the modeling and theory, F.S.R., T.P.J.K., G.D., H.S., S.K.S., and R.M. advised the study and all authors contributed to the manuscript writing.

## Supporting information

Supporting Information

## Data Availability

The data that support the findings of this study are available from the corresponding author upon reasonable request.
