# Supplementary material for: Hierarchical Protofilament Intertwining Rules the Formation of Mixed‐Curvature Amyloid Polymorphs
Source: Adv Sci (Weinh). 2024 Jun 20;11(32):2402740. doi: 10.1002/advs.202402740 (PMC11348146; doi:10.1002/advs.202402740)
Supplement: Supplementary file 1 — Supporting Information [file ADVS-11-2402740-s001.docx]

Supporting Information

Hierarchical Protofilament Intertwining rules the Formation of Mixed-Curvature Amyloid Polymorphs

Jiangtao Zhou, Salvatore Assenza, Meltem Tatli, Jiawen Tian, Ioana M. Ilie, Eugene L. Starostin, Amedeo Caflisch, Tuomas P. J. Knowles, Giovanni Dietler, Francesco S. Ruggeri, Henning Stahlberg, Sergey K. Sekatskii*, and Raffaele Mezzenga*

J. Zhou, G. Dietler, S.K. Sekatskii

Laboratory of Physics of Living Matter, Institute of Physics, Ecole Polytechnique Fédérale de Lausanne (EPFL), CH-1015 Lausanne, Switzerland

E-mail: [serguei.sekatski@epfl.ch](mailto:serguei.sekatski@epfl.ch)

J. Zhou, R. Mezzenga

Department of Health Sciences and Technology, ETH Zurich, Zurich, Switzerland

E-mail: raffaele.mezzenga@hest.ethz.ch

S. Assenza

Departamento de Física Teórica de la Materia Condensada, Universidad Autónoma de Madrid, 28049 Madrid, Spain

S. Assenza

Condensed Matter Physics Center (IFIMAC), Universidad Autónoma de Madrid, 28049 Madrid, Spain

S. Assenza

Instituto Nicolás Cabrera, Universidad Autónoma de Madrid, 28049 Madrid, Spain

M. Tatli, J. Tian, H. Stahlberg, S. K. Sekatskii

Laboratory of Biological Electron Microscopy, Institute of Physics, SB, EPFL, and Dep. of Fund. Microbiol., Faculty of Biology and Medicine, UNIL, Rt. de la Sorge, 1015 Lausanne, Switzerland

I.M. Ilie

van’t Hoff Institute for Molecular Sciences, University of Amsterdam, P.O. Box 94157, 1090 GD Amsterdam, The Netherlands

I.M. Ilie

Amsterdam Center for Multiscale Modeling (ACMM), University of Amsterdam, P.O. Box 94157, 1090 GD Amsterdam, The Netherlands

E. L. Starostin

Department of Civil, Environmental & Geomatic Engineering, University College London, Gower Street, London WC1E 6BT, UK

A. Caflisch

Department of Biochemistry, University of Zürich, Zürich CH-8057, Switzerland

T. P. J. Knowles

Department of Chemistry, University of Cambridge, Lensfield Road, Cambridge CB2 1EW, UK

F.S. Ruggeri

Laboratory of Organic Chemistry, Stippeneng 4, 6703 WE, Wageningen University & Research, the Netherlands

F.S. Ruggeri

Physical Chemistry and Soft Matter, Stippeneng 4, 6703 WE, Wageningen University & Research, the Netherlands

R. Mezzenga

ETH Zurich, Department of Materials, 8093 Zurich, Switzerland

**Supplementary text**

1. **Evolution of amyloid fibril morphology.**

A collection of AFM images recapitulating the formation process of chiral fibrils is shown in **Fig. S2**. Before incubation, the initiating protein shows monomeric or oligomeric forms. After one hour of incubation, early single protofilaments are observed, with a height of 1 nm and no significant height fluctuations. In the following hours, we observed homogeneous chiral protofibrils with fluctuating height profiles over the evolution process. The average height of these chiral fibrils shows multimodal peaks in the distribution at different incubation times (**Fig. S3**). Moreover, these peaks show a hierarchical distribution (**Fig. 1d**) as a function of incubation time.

1. **Characterization of aggregation kinetics and structural transition.**

The structural characterization of insulin aggregates during the incubation was carried out by using three independent bulk analytic approaches.

Firstly, the aggregation kinetics was monitored by using Thioflavin T (ThT) fluorescence (**Fig. 1a**). ThT signal was measured for 12 hours of incubation and data were fitted with a sigmoidal model as described in the methods section. An initiating lag phase^[1]^ of 4 hours was observed, followed by the growth phase, until the ThT signal reached a plateau after 10 hours of incubation.

Secondly, circular dichroism (CD) was performed to characterize the secondary structural transition. According to the CD spectra (**Fig. S1a**), the insulin solution after 1-hour incubation shows a predominant α-helix structure, whose signal gradually decreases as a function of incubation time. However, a dominant cross-β sheet structure, showing strong absorption at 218 nm, was observed after 8-10 hours of incubation.

Lastly, the structural transition of insulin aggregates was further characterized by infrared nanospectroscopy. The spectrum at 0h shows the co-existence of α-helix and β-sheet conformation in the initiating natively folded monomers. The conformational transition from α-helix (blue) to β-sheet (red) in the amide I in the absorption spectra (**Fig. S1b**) shows the rapid increase of β-sheet content after 4 hours. The spectra were processed according to previous methods^[2]^, and sub-bands in the amide I region (1600-1700 cm^-1^) were revealed by multiple Gaussian fitting^[3]^, including helical and turn-like (1648-1685 cm^-1^) structure, β-sheet and antiparallel β-sheet (1610-1640 and 1685-1695 cm^-1^) structure and random coil (1640-1650 cm^-1^) structure. The transition from α-helix to β-sheet content can be noticed throughout the aggregation process, and the β-sheet component starts to dominate the secondary structure of the aggregate after 6-8 hours of incubation.

1. **Nucleation events during the aggregation in the early stages revealed by AFM.**

In addition to the snapshots of the intertwining of protofilaments and protofibrils (**Fig. 2b** and **S2**), AFM also captured some key aggregation steps, such as primary and secondary nucleation (**Fig. S4)**. An example of primary nucleation, namely the assembly of a monomer and a dimer (with a height of 1.5 nm), is shown in **Fig. S4a**, together with an individual monomer for comparison (height of 1 nm). The secondary nucleation of an early protofilament is shown in **Fig. S4b**, where the height of a protofilament (1 nm) is increased upon the attachment of a monomer. **Fig. S4c** shows two branches of fibrils growing from a preformed chiral protofibril, providing an example of the nucleation among protofibrils and the intertwining mechanism of higher-ordered protofibril formation.

1. **Insulin fibrillization process at a large ionic strength.**

To check the effect of the strength of fibril-fibril attraction on chiral fibril formation, we repeated the experiment (**Fig. S5**) in the presence of salt (150 mM NaCl) to minimize the electrostatic repulsion among the fibrils, thus increasing the attraction between the positively charged protofilaments and protofibrils^[4]^. A large number of massive fibril bundles were formed within a short time showing much thicker (several tens of nanometer) morphologies. This suggests that the condition without NaCl provides an appropriate modulation force of surface interaction that is critical for a gentle fibril-fibril interaction and therefore the possibility for forming multi-stranded fibrils with complex packing schemes^[5]^.

**Fig. S1 Secondary structural transition during insulin fibrillization**


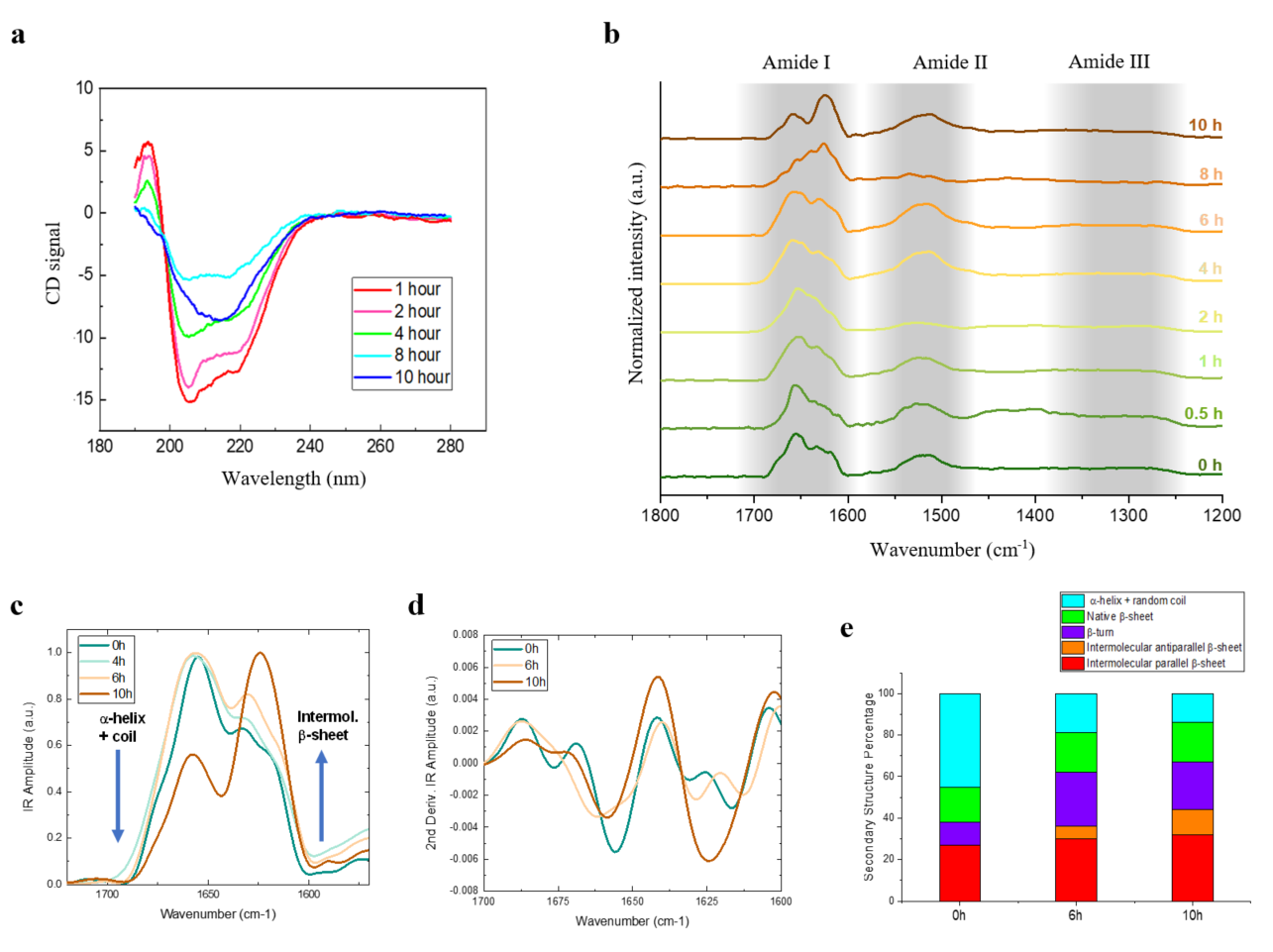


**Fig. S1. Secondary structural transition during insulin fibrillization.** **(a)** CD spectra of insulin solution incubated for 1, 2, 4, 8, and 10 hours. The dips on the spectrum at 208 and 220 nm (α-helical conformation) show a gradual shrinking in the first 8 hours and finally transfer to 218 nm (β-sheet content) in the following 2 hours. **(b)** Localized FTIR absorption spectra of the thin film of aggregates from 0 to 10 hours incubation in the wavenumber range 1200-1800 cm^-1^. The band of amides I, II, and III are indicated with a grey background. (**c-d**) the amide I and the second derivatives of several representative spectra with different incubation time. (**e**) Secondary structure conformation of the representative spectra from panel c-d, showing a clear trend of α-helix to β-sheet transition.

**Fig. S2 Observation of insulin fibrillization from monomer to mature fibril**


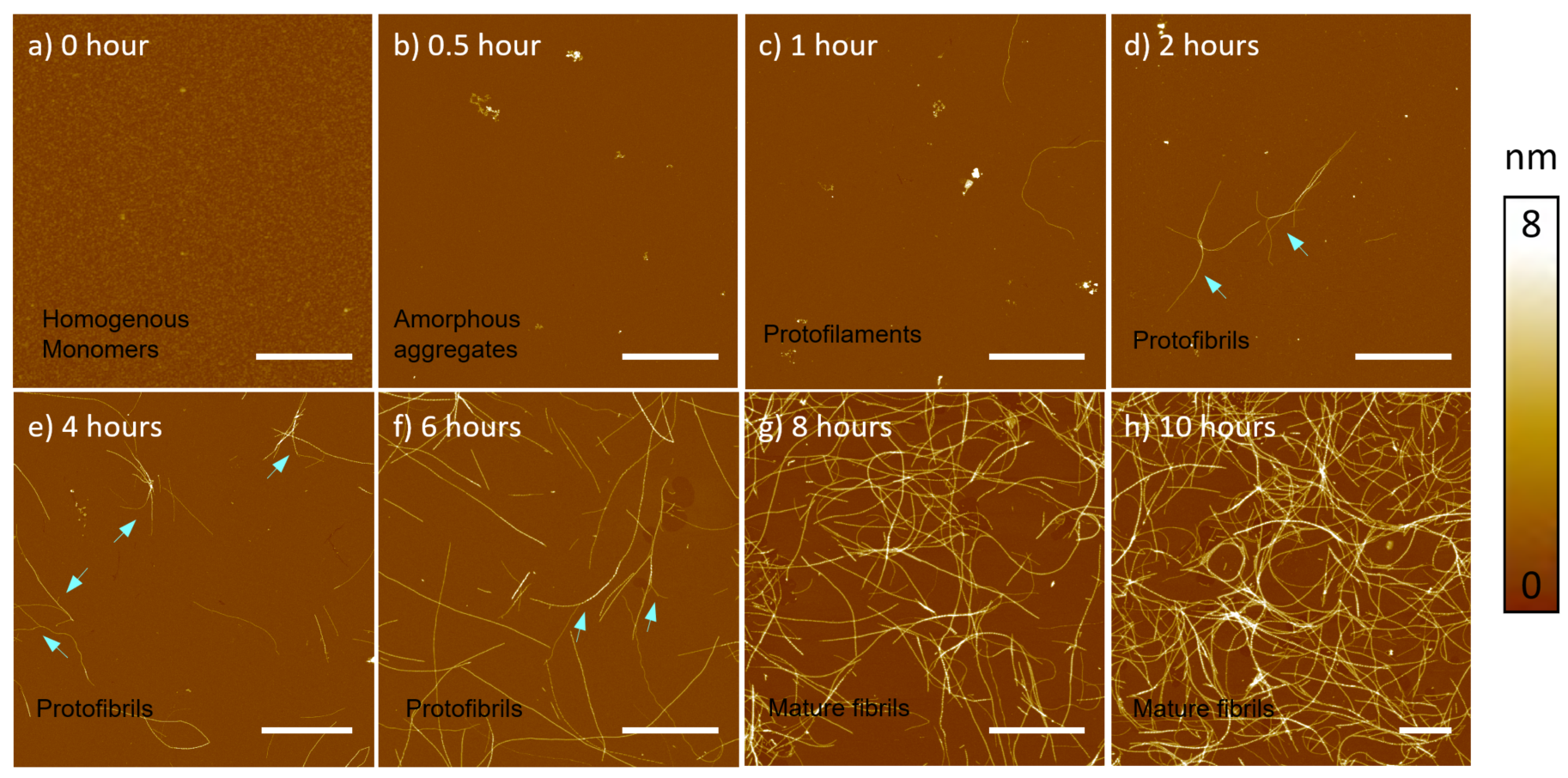


**Fig. S2 Observation of insulin fibrillization from monomer to mature fibril.** **(a-h)** AFM images of insulin self-assemblies incubated for 0, 0.5, 1, 2, 4, 6, 8, and 10 hours. These AFM images show **(a)** homogeneous monomeric insulin; **(b)** amorphous pre-fibrillar aggregates; **(c-f)** protofilaments and protofibrils; **(g-h)** mature fibrils. The fibril-fibril interaction among protofilaments and protofibrils are indicated by arrows which mainly occurred in the lag and growth phases. Scale bars are 2 µm.

**Fig. S3 Statistical analysis of the average height of insulin aggregates**


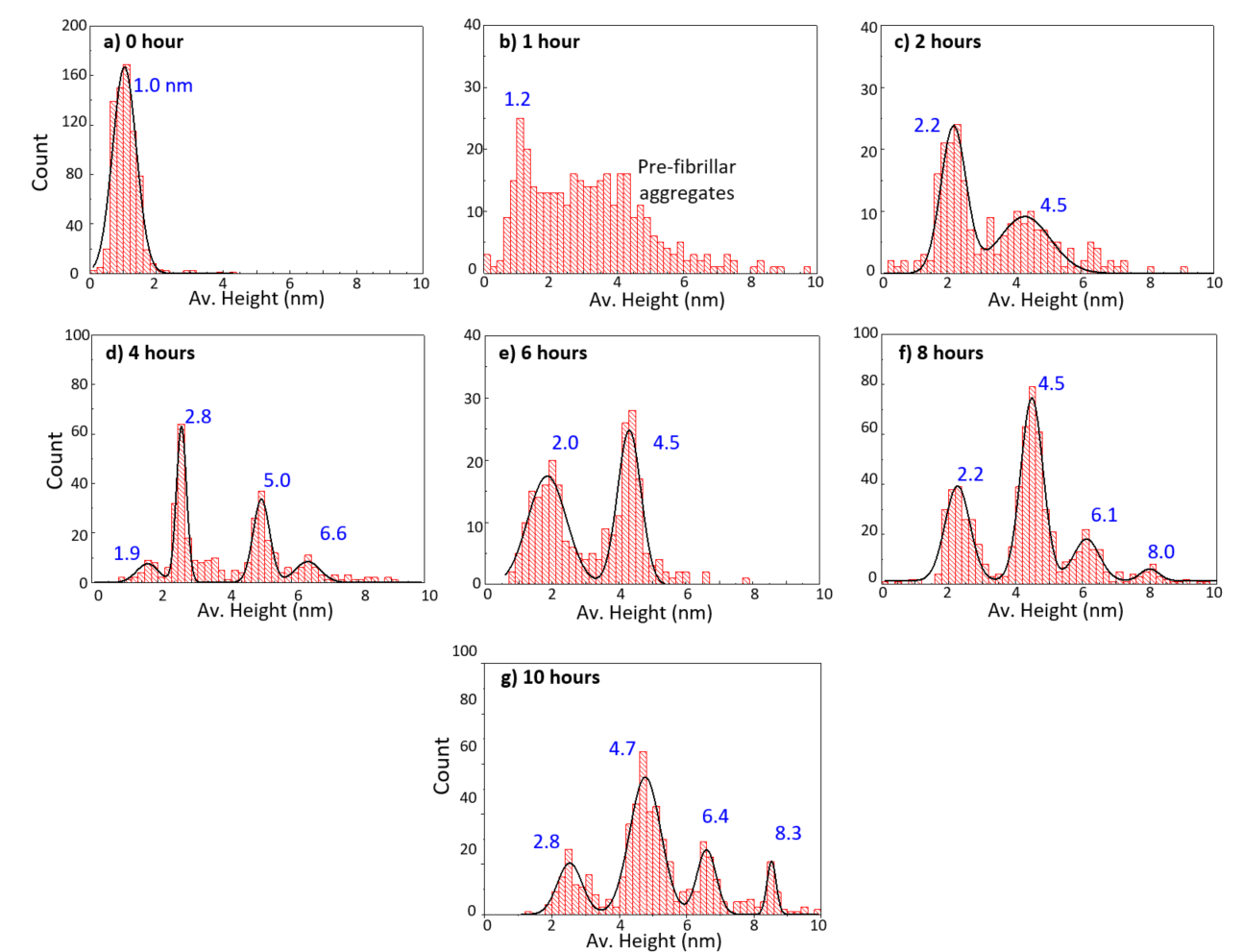


**Fig. S3 Statistical analysis of the average height of insulin aggregates.** (**a)** The height distribution of insulin proteins before incubation shows an average height of 1.0 nm, indicating homogeneous monomeric proteins under an acidic condition^1^. (**b**) Height distribution of protein aggregates incubated for 1 hour. The distribution of the average height of protofilaments is located at 1.2 nm, but another cluster of height distributions (1-8 nm) was found because of the existence of amorphous pre-fibrillar aggregates (Fig. S2b-c). (**c-g**) The height distributions of protofibrils and fibrils obtained after 2-10 hours of incubation show a multimodal shape with multiple peaks, indicating the presence of distinct families.

**Fig. S4 Visualization of fibrillization steps in the early stages of aggregation**


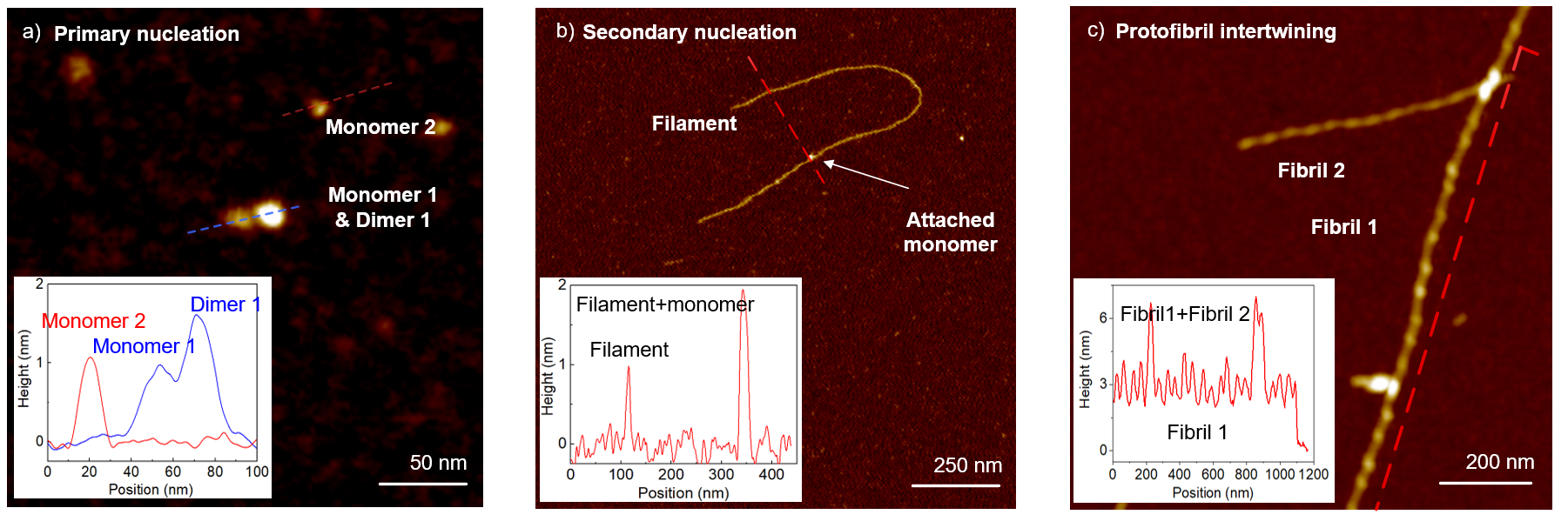


**Fig. S4 Visualization of fibrillization steps in the early stages of aggregation. (a)** An example of primary nucleation of monomers and dimers after 1-h incubation. The inset shows the height profile of monomer 2 (red) and the assembly of monomer and dimer (blue). **(b)** An example of secondary nucleation involving a monomer and a protofilament observed after 1-h incubation. The inset shows the height profile traced along the red dashed line. **(c)** Fibrillar aggregates obtained from the 4h-incubated solution show the intertwining of one protofibril with another protofibril to form a higher-ordered fibril. The inset shows the height profile along the red dashed line.

**Fig. S5 Insulin fibrillization at high ionic strength**


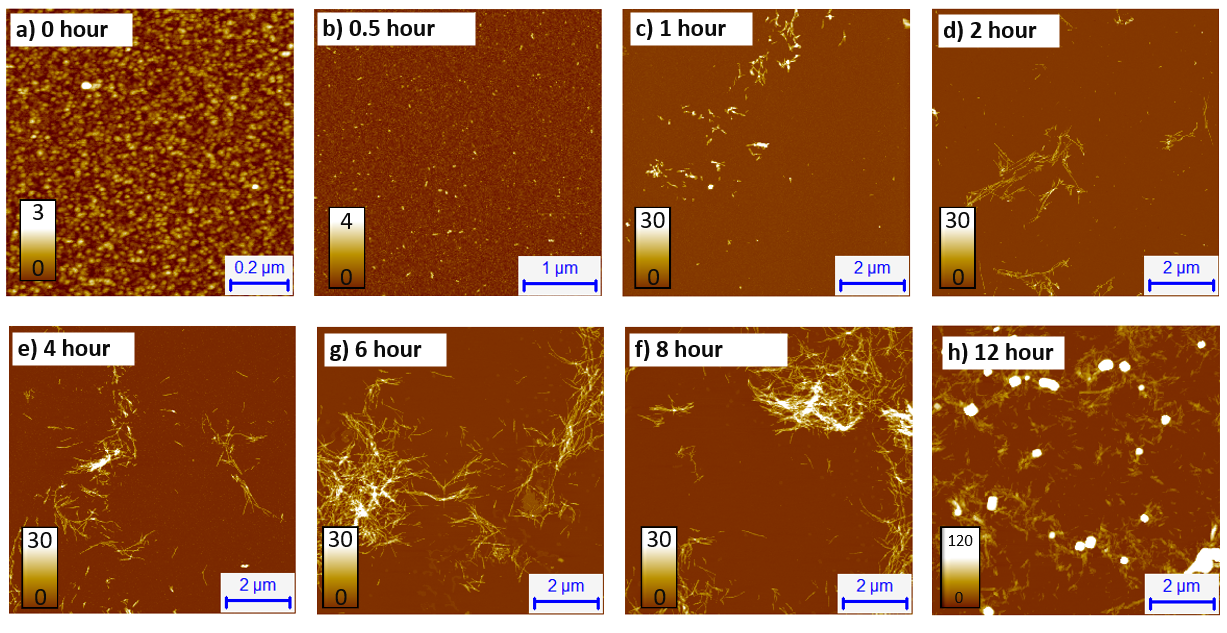


**Fig. S5 Insulin fibrillization at high ionic strength. (a-h)** AFM images of insulin aggregates obtained in the presence of 150mM NaCl after 0, 0.5, 1, 2, 4, 6, 8, and 12 hours of incubation, respectively. Apart from the addition of salt, the same conditions as in the other experiments of this work were considered (i.e. a buffer of 25 mM HCl, 150 mM NaCl, pH 1.6). Compared to the case without NaCl, these fibrils were much shorter with no clear fluctuating morphology and became short fibril bundles in the later stages of aggregation.

**Fig. S6 Illustration of AFM convolution effect while scanning fibrils**


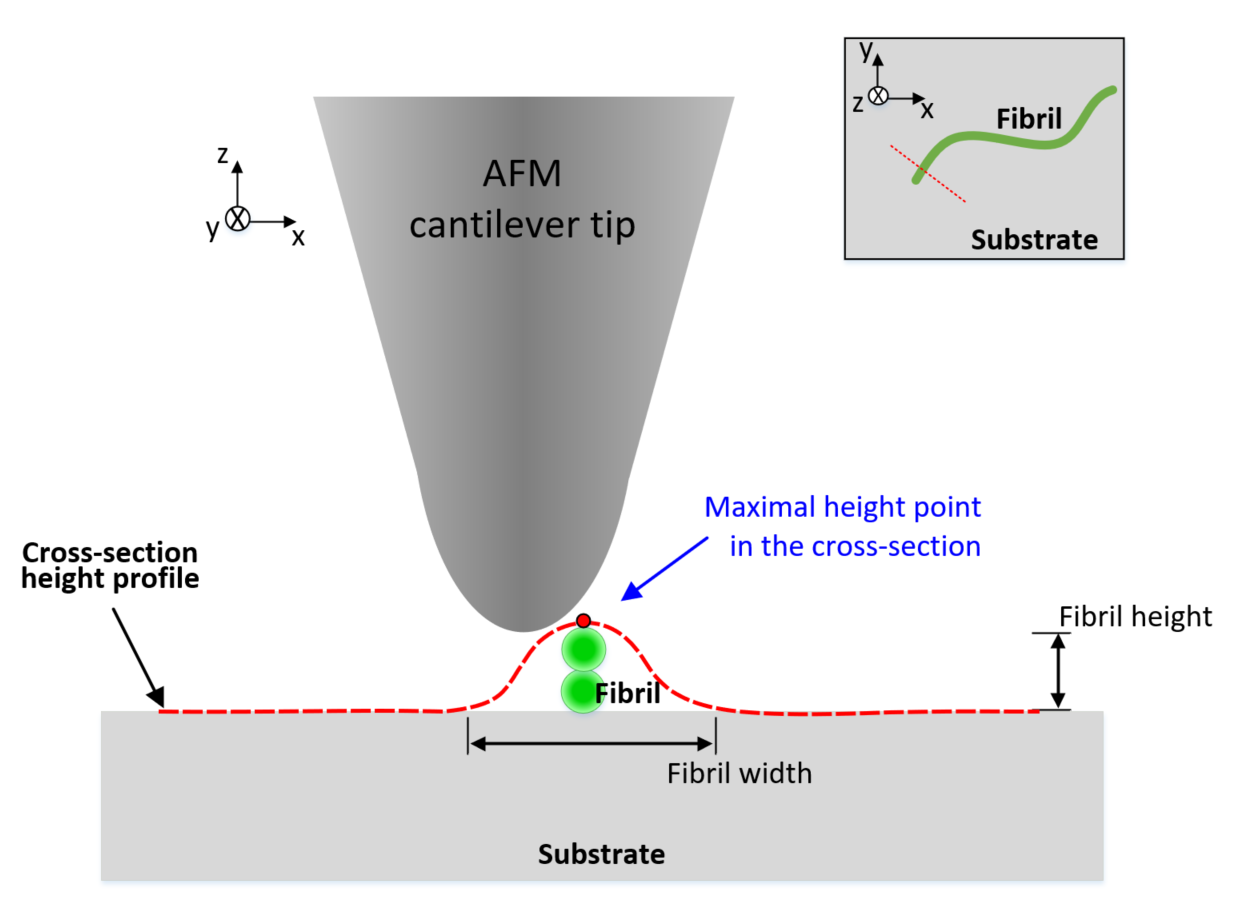


**Fig. S6 Illustration of AFM convolution effect while scanning fibrils.** Since the diameter of amyloid fibrils is as small as few nanometers (usually in the range of 1-9 nm) that is comparable to the radius of AFM tip apex, the fibril profile in the cross-section of fibril contour length (red curve) does not capture the fibril morphology with good accuracy. This convolution effect varies with AFM tip radius, shape, and cone angle, as well as sample diameter and operation mode. In this experiment, the widths of fibrils were measured in the range of 20-50 nm. However, AFM shows angstrom-level sensitivity in the z-direction, thus the maximal height in the cross section gives accurate information about the amyloid fibril morphology in the vertical direction. Therefore, tracing the maximum height along the fibril (Fig. 2c) allows for the recording of reliable geometrical details of fibril configurations.

**Fig. S7 Assessment of average height and maximal height of insulin fibrils**


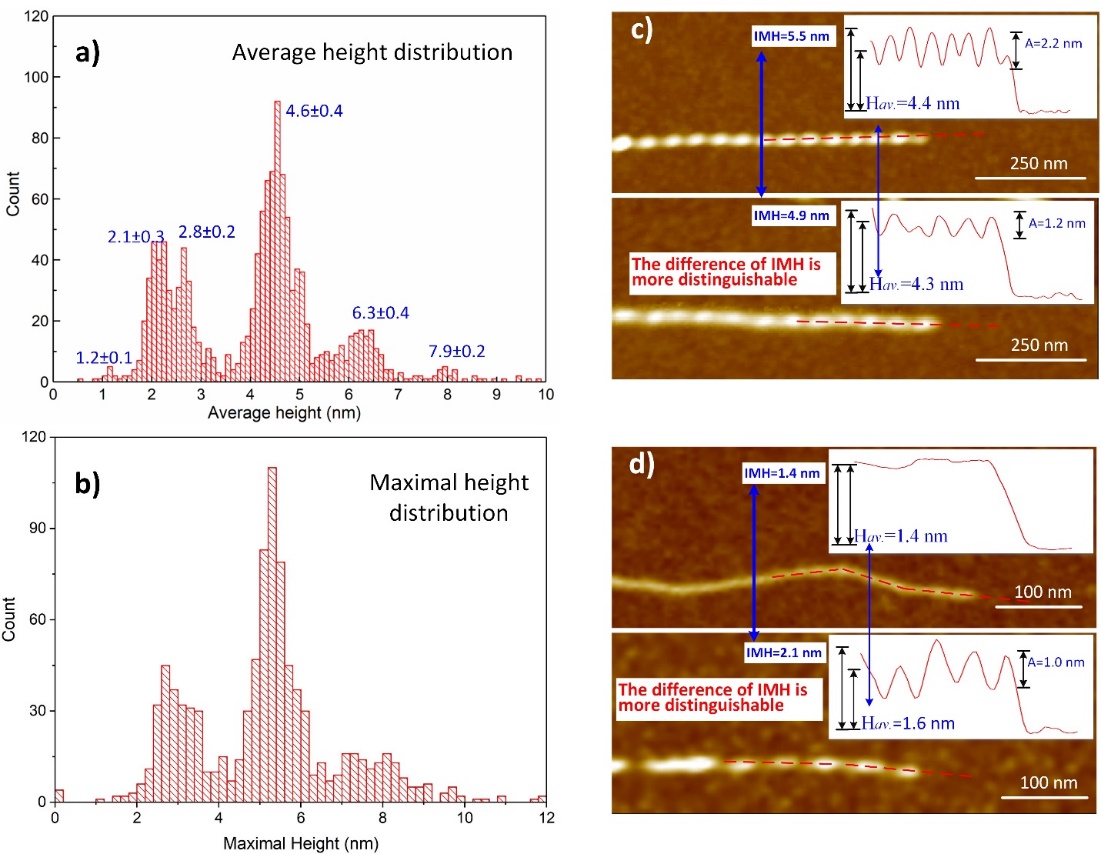


**Fig. S7 Assessment of average height and maximal height of insulin fibrils. (a)** Histogram of fibril average height acquired by averaging heights along the ridge of each amyloid fibril. **(b)** Histogram of maximal height acquired by averaging the height of the peaks on the fibril fluctuating height profile. This histogram shows only three main peaks. **(c)** Two chiral fibrils with different IMH and amplitudes imply distinct numbers of protofilaments and configurations but show a similar average height. **(d)** A protofilament shows an average height similar to another chiral fibril, whereas its IMH and amplitude are different. These two examples (c-d) epitomize the advantages derived from employing the IMH over the average height to characterize the features of these hierarchical chiral fibrils.

**Fig. S8 Possible configurations of fibrils with IMH 4 nm.**


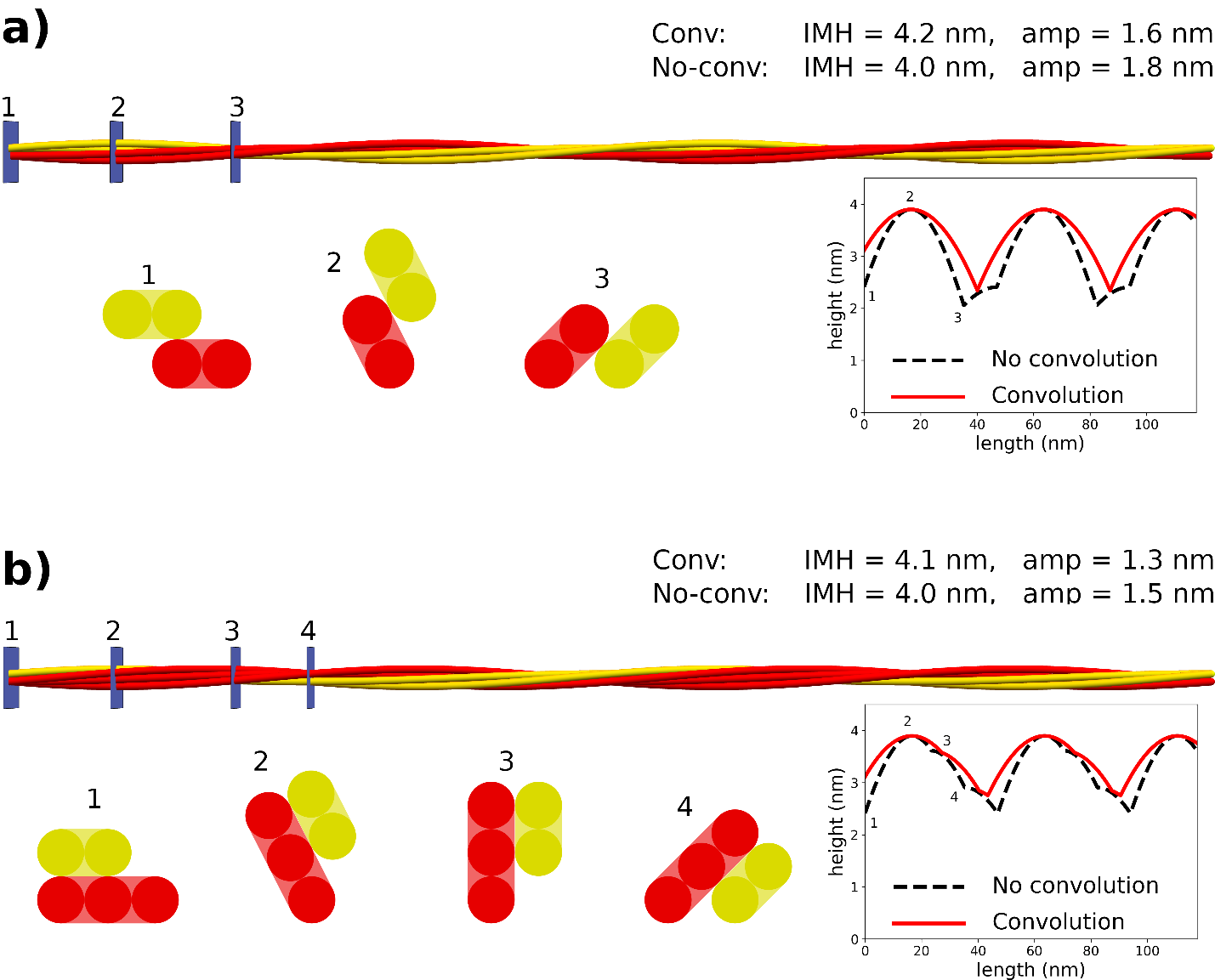


**Figure S8. Possible configurations of fibrils with IMH 4 nm.** Relaxing the assumption of the existence of a vertically-aligned arrangement, it is possible to reconcile the 4 nm peak of the IMH distribution in Fig.2e with the proposed theoretical construction. a) Fibril obtained by stacking two $2_{[1]}$ protofibrils in their horizontal conformation and shifted by the size of a protofilament $b$, so that they are attached only via one protofilament. Starting from this, we combine them by setting $k=1$, which ensures maintaining the shape of the section along the fibril. Since the maximum height (corresponding to configuration 2 and equal to ($\sqrt{5}+1)b$) and minimum height (configuration 3 and equal to $(1+1/\sqrt{2})b$) correspond to configurations in which the protofibrils are not horizontally or vertically adsorbed, this packing mechanism corresponds to non-integer indices in our classification and is coded as ${(\sqrt{5}+1)}_{[\sqrt{5}-1/\sqrt{2}]}$ b) Same as a) but combining a $2_{[1]}$ and a $3_{[2]}$ protofibril. The section is obtained by aligning the protofilaments of $2_{[1]}$ to two protofilaments of the three-stranded ribbon. Again, we set $k=1$. This packing mechanism is coded by ${(\sqrt{5}+1)}_{[\sqrt{5}-1]}$according to our classification, corresponding to a minimum height $2b$ in configuration 1 and to a maximum height as in panel a). In both a) and b), for key points along the fibrils the corresponding sections are depicted, and the corresponding points on the AFM profiles are labelled accordingly. Particularly, the maxima (sections labelled as 2) correspond to sections in which the most extreme protofilaments of the intertwining protofibrils are aligned in the vertical direction. The values of IMH and amplitude are reported for both the convoluted and non-convoluted profiles.

**Fig. S9 Evaluation of the pitch of chiral fibrils.**


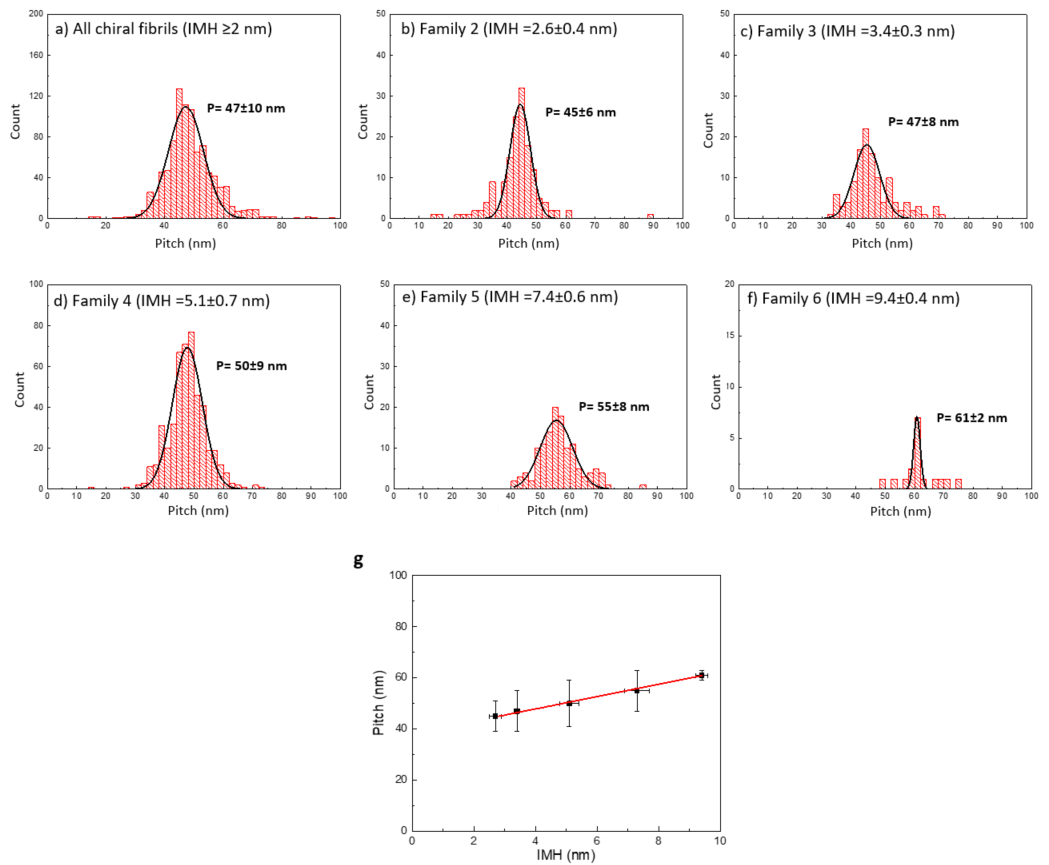


**Figure S9. Evaluation of the pitch of chiral fibrils.** **(a)** Histogram of pitch ($P$) distribution of all chiral fibrils with an IMH height of more than 2 nm. The pitch is the distance between consecutive peaks along the fluctuating height profile, which is half of the period ($L$) of the chiral fibrils. **(b-f)** Distribution of the pitch of chiral fibrils from each IMH family, where there is only one dominant peak in each family. **(g)** Average pitch in each fibril family shows a linear dependence on IMH.

**Fig. S10 Amplitude assessment of chiral fibrils from family 7**


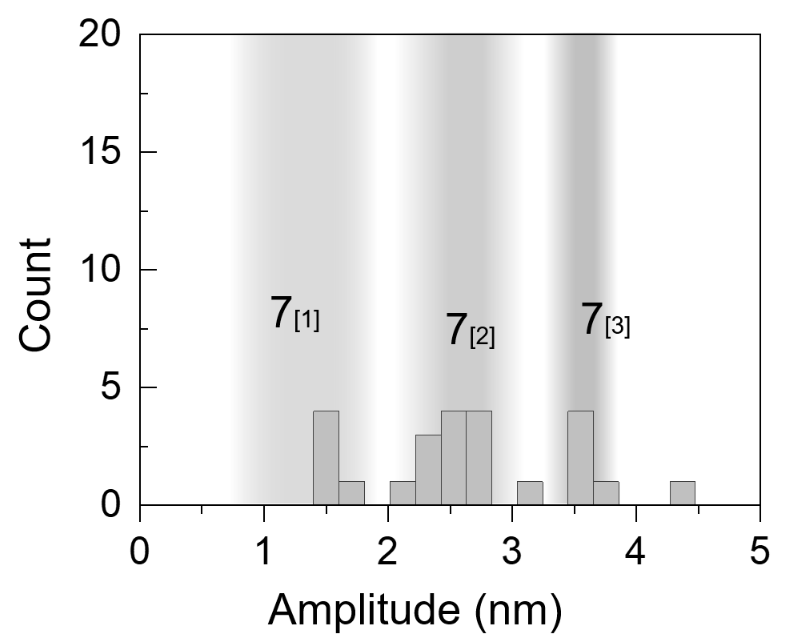


**Figure S10. Amplitude assessment of chiral fibrils from family 7.** The amplitude distribution of chiral fibrils from classified IMH family 7 with IMH of 9.4±0.4 nm. Regions shaded in grey refer to the IMH of fibrils with 1, 2, and 3 protofilaments.

**Fig. S11 Cryo-EM images and initial 3D models of four distinct polymorphs**


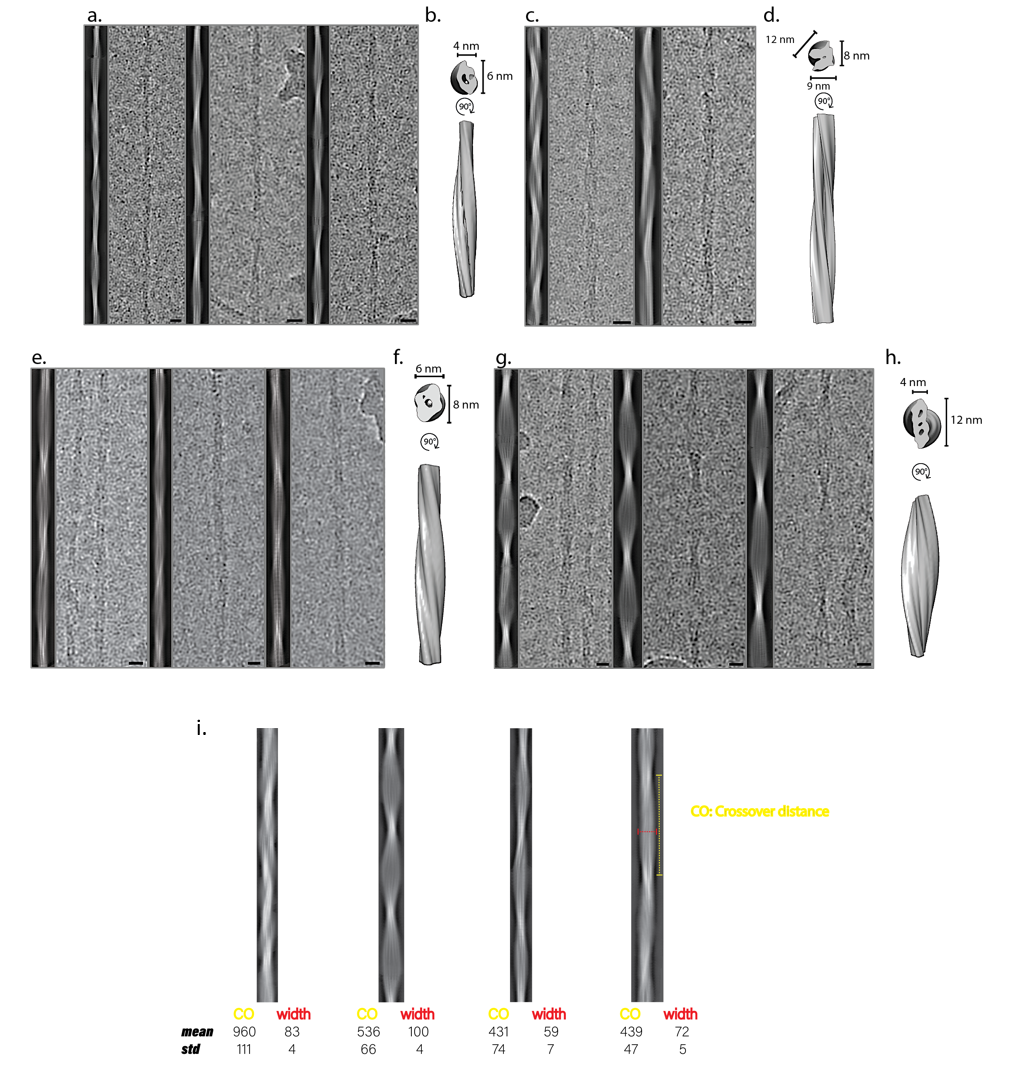


**Fig. S11 Cryo-EM images and initial 3D models of four distinct polymorphs.** a) During the early stages of aggregation, the filament type Thin1 dominates and is characterized by its narrow width and significant variability in crossover distances, as evident from reconstituted fibrils and micrographs placed alongside each other. b) An averaged 3D initial model of Thin1 filament in the cross-section. c) In the later stages of assembly, the highly-ordered regular thick filament becomes the most prevalent, formed by the largest number of protofilaments, as indicated by the dimensions of reconstituted fibrils and their corresponding 3D model. d) Compared to other polymorphs, the 3D initial model of the thick fibril appears to be more densely packed and does not show any visible gaps or openings between its protofilaments. e) Thin2 has a slightly larger width than Thin1. f) The Thin2 3D model also suggests a gap from the top cross-section which is more uniform and symmetrical compared to Thin1. It may have 2-fold or pseudo-2-fold symmetry, as revealed by the corresponding 2D classes (Fig. S12). g) The twisted ribbon morphology has a consistent width in its thickest section, implying the presence of a similar number of protofilaments, although there are variations in cross-over distances. h) The 3D model of the twisted ribbon morphology reveals a loosely assembled arrangement of protofilaments. The presence of gaps in both twisted ribbons and mixed-curvature fibrils suggests that these gaps might be an intrinsic property of anisotropic protofilaments after assembling. The scale bars are 8 nm. i) The quantified crossover distances and widths of the identified four d polymorphs.

**Fig. S12 A closer inspection at large box size of polymorph 2D classes**


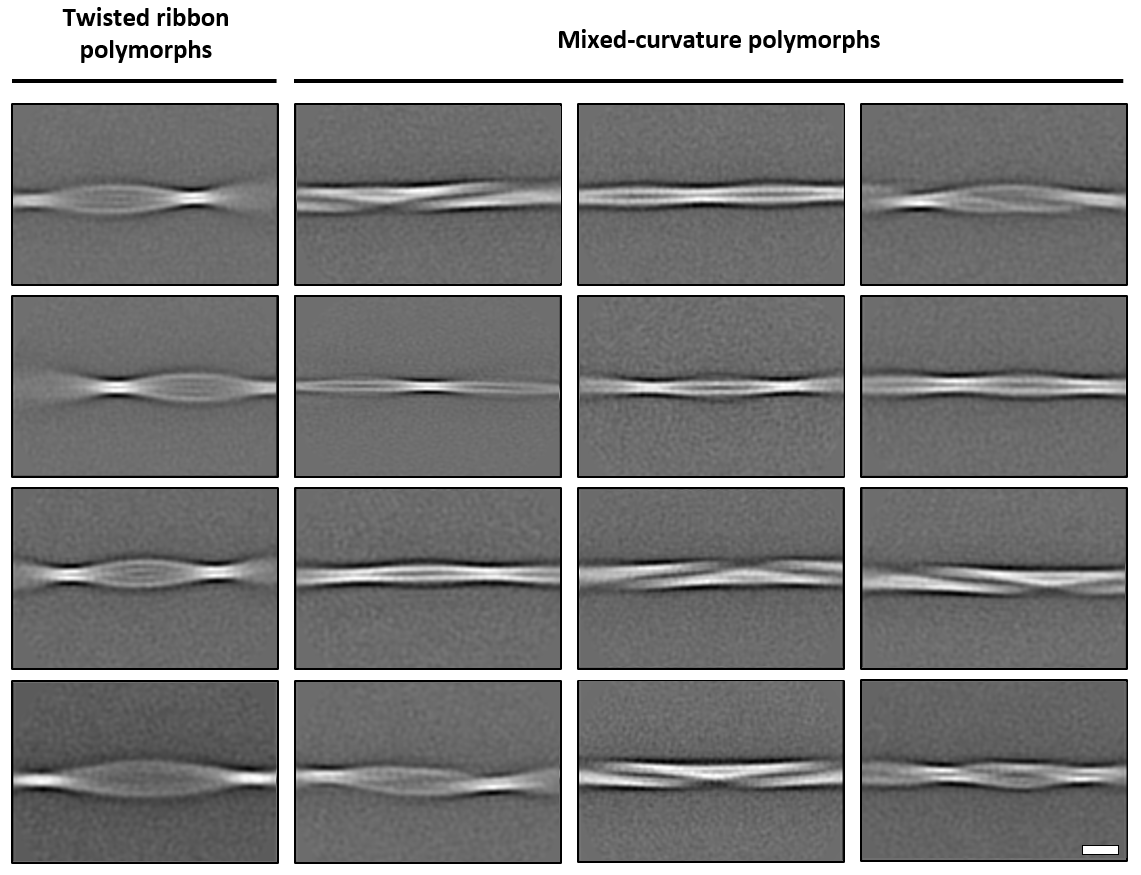


**Fig. S12 A closer inspection at large box size of polymorph 2D classes.** The initial 2D classes, generated with large box size, revealed a vast heterogeneity of different polymorphs. Mixed-curvature polymorphs were found to exhibit greater variations. The scale bar is 10 nm.

**Fig. S13 A closer inspection at small box size of polymorph 2D classes**


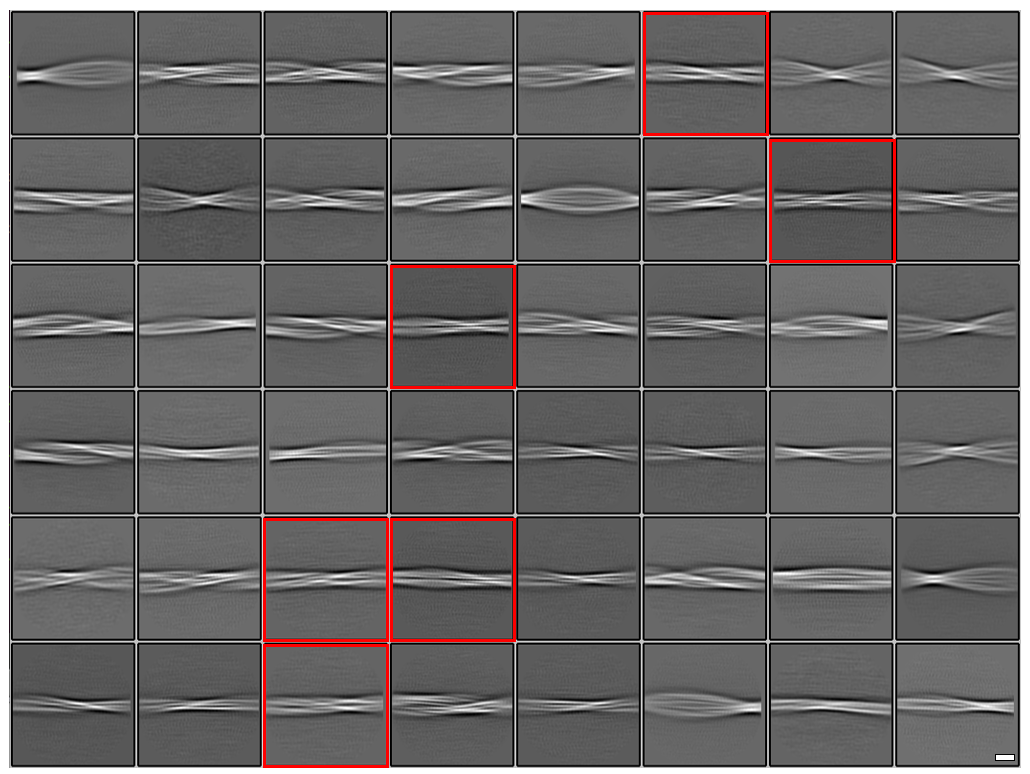


**Fig. S13 A closer inspection of the small box size of polymorph 2D classes.** By using a smaller box size to focus on specific regions, we were able to observe the individual packing of protofilaments and identify a new polymorph, Thin2, which appears to have a 2-fold symmetry. The new polymorph was highlighted in red squares in the images. The scale bar is 10 nm.

**Fig. S14 Examples of intertwining between early protofibrils**


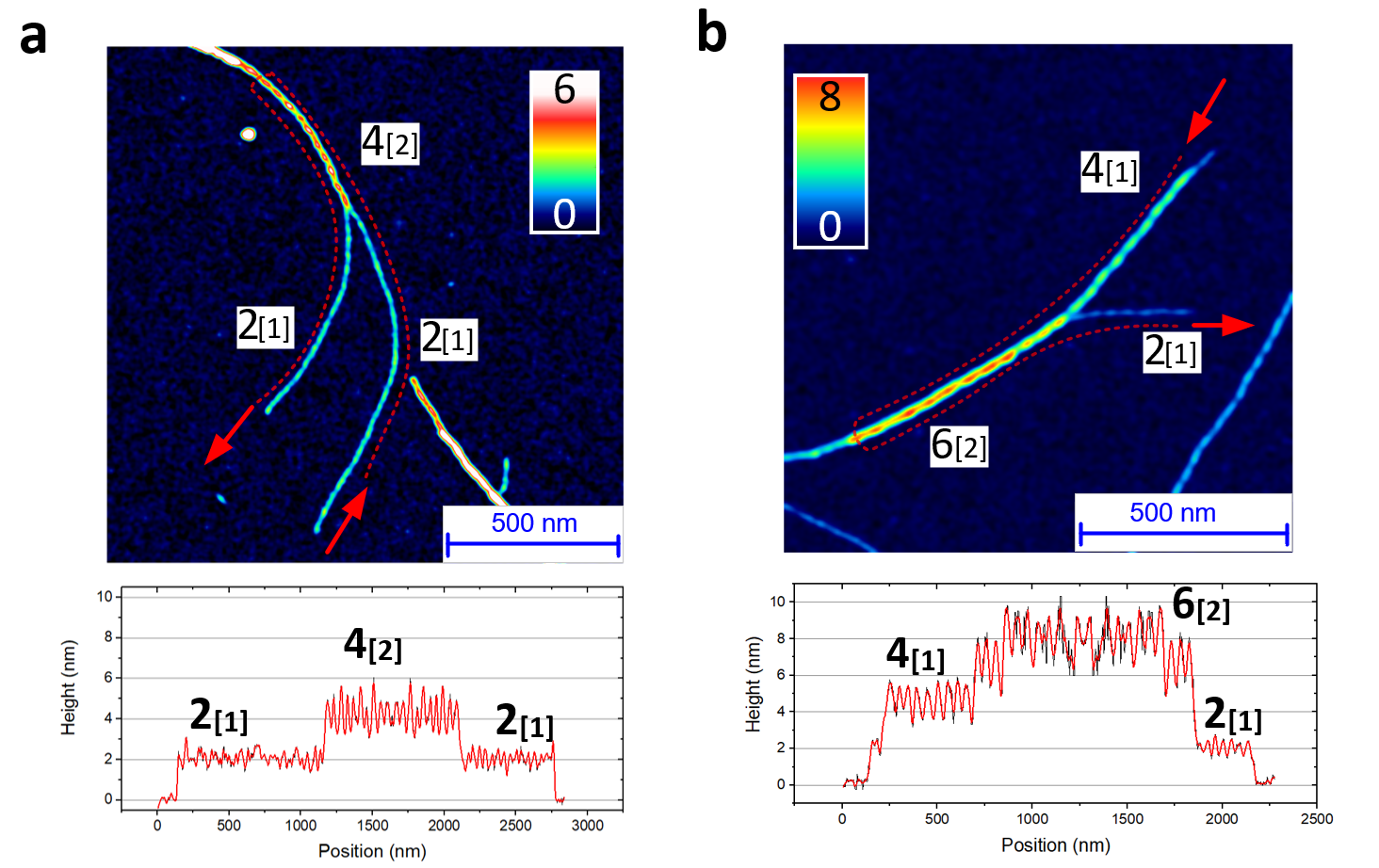


**Fig. S14 Examples of intertwining between early protofibrils.** (a) AFM snapshot showing the intertwining of two identical early protofibrils $2_{[1]}$ into the higher-ordered protofibril $4_{[2]}$ with their height fingerprints of each protofibril for the classification of protofibrils. (b) AFM snapshot showing the combination of two protofibrils from families $2_{[1]}$ and $4_{[1]}$, identified according to their IMH and amplitude, to form a higher-ordered fibril from $6_{[2]}$ family, following our proposed intertwining rule: $4_{[1]} \oplus2_{\left[ 1 \right]} \to6_{\left[ 2 \right]}$. The inset in each figure indicates the height profile along the red dashed line following the direction of the red arrows.

**Fig. S15. Schematic representation of twisted ribbons and mixed-curvature polymorphs**


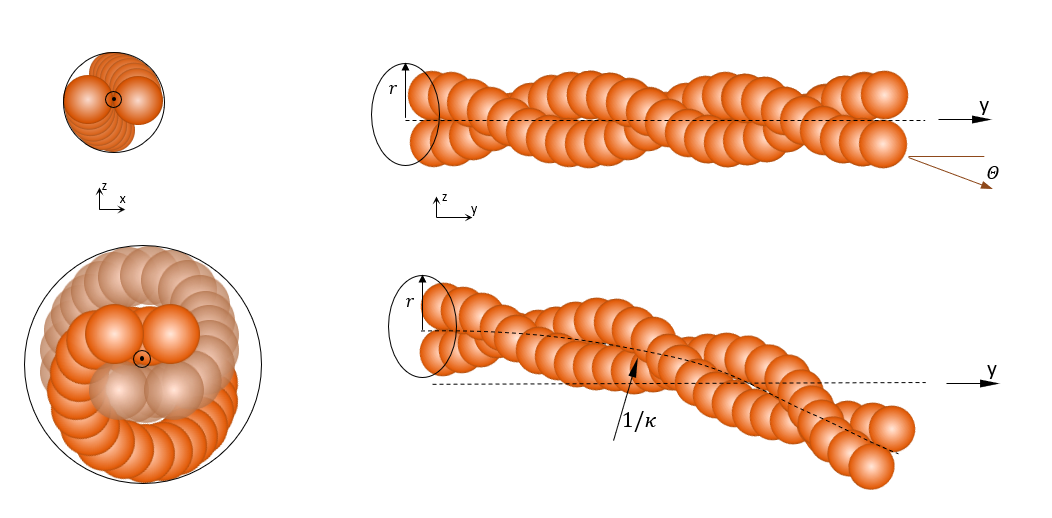


**Fig. S15. Schematic representation of twisted ribbon polymorphs and mixed-curvature polymorphs.** The schemes represent a twisted protofibril $2_{[1]}$ and a bending twisted ribbon $2_{[1]}$ in the mixed-curvature polymorphs $4_{[2]}$, as observed in the AFM snapshot in Figure 4b. In this presented scheme, $\theta$ represents the twist angle in the twisted ribbon and $1/\kappa$ refers to the radius of the bending curvature in the mixed-curvature fibril.

**Fig. S16. Theoretical methods**


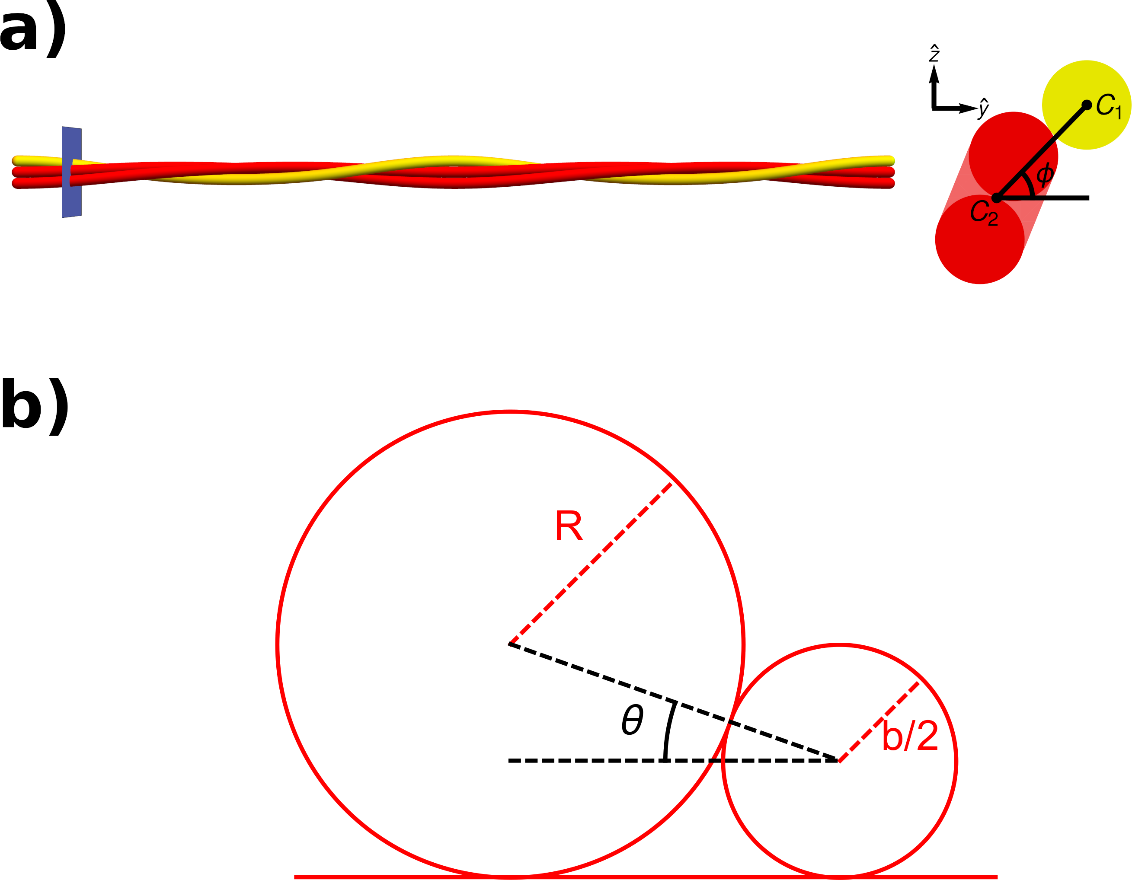


**Fig. S16. Theoretical methods.** (a) Structure of $3_{[1]}$ (left) and section corresponding to the intersection with the blue plane, where the phase $\phi$ is defined (right). (b) Geometrical construction for the estimation of the apparent width of a protofilament due to the tip convolution.

**Fig. S17 Calculated AFM profiles for a twisted ribbon composed of two protofilaments**


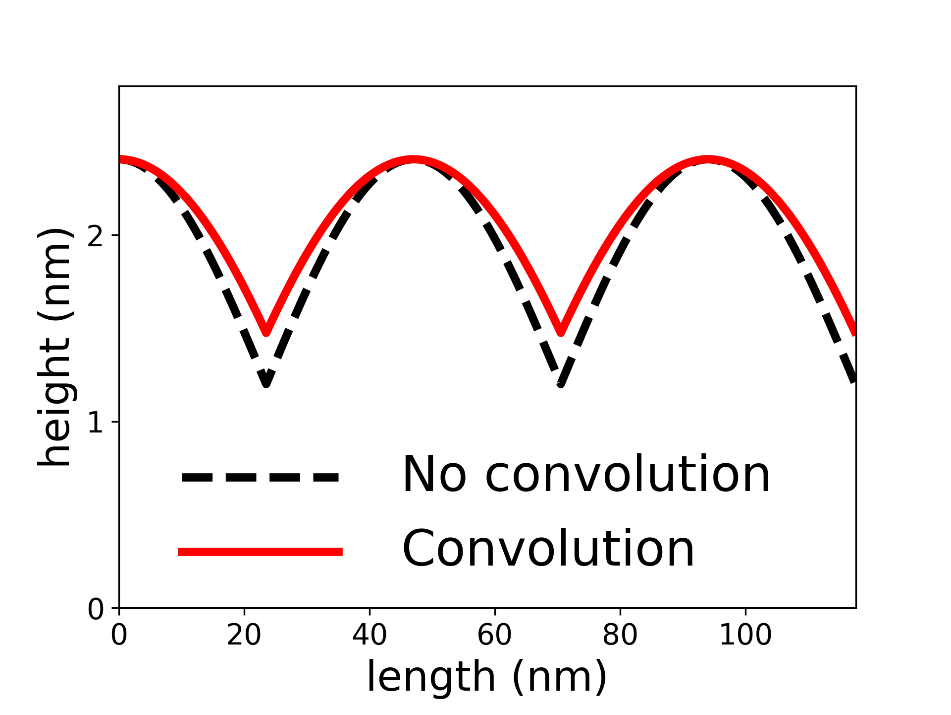


**Fig. S17. Calculated AFM profiles for a twisted ribbon composed of two protofilaments**. At difference with the mixed-curvature cases, for a twisted ribbon the convolution of the tip does not alter the qualitative features of the height profile.

**Fig. S18. Theoretical modeling of an outlier**


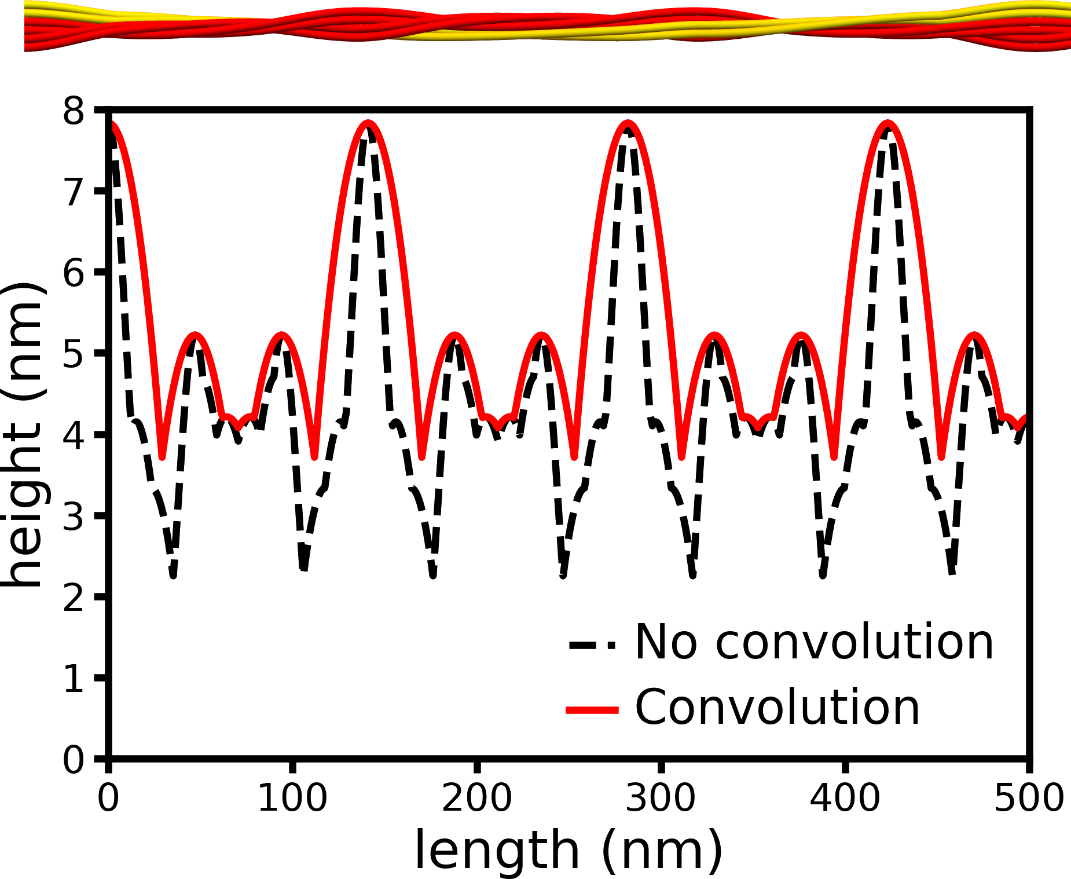


**Fig. S18. Theoretical modeling of an outlier.** The structure (top) and theoretical AFM profile (bottom) obtained for the fibril reported in Fig.5a in the main text. The dashed black line and continuous red line correspond to the “true” profile and to the convoluted case with $R=90$ nm, respectively. The fibril was built according to the combination rule $4_{[2]}\oplus_{3/2}2_{[1]}$, corresponding to $k=3$.

**References**

[1] a) L. Nielsen, R. Khurana, A. Coats, S. Frokjaer, J. Brange, S. Vyas, V. N. Uversky, A. L. Fink, *Biochemistry* **2001**, *40*, 6036–6046; b) S. Campioni, G. Carret, S. Jordens, L. Nicoud, R. Mezzenga, R. Riek, *J. Am. Chem. Soc.* **2014**, *136*, 2866–2875.

[2] F. S. Ruggeri, G. Longo, S. Faggiano, E. Lipiec, A. Pastore, G. Dietler, *Nature Communications* **2015**, *6*, 7831.

[3] J. Seo, W. Hoffmann, S. Warnke, X. Huang, S. Gewinner, W. Schöllkopf, M. T. Bowers, G. von Helden, K. Pagel, *Nature Chemistry* **2016**, DOI 10.1038/nchem.2615.

[4] G. Meisl, X. Yang, C. M. Dobson, S. Linse, T. P. J. Knowles, *Chem. Sci.* **2017**, *8*, 4352–4362.

[5] a) T. P. J. Knowles, M. J. Buehler, *Nature Nanotechnology* **2011**, *6*, 469–479; b) R. Paparcone, S. W. Cranford, M. J. Buehler, *Nanoscale* **2011**, *3*, 1748–1755; c) J. L. Jiménez, E. J. Nettleton, M. Bouchard, C. V. Robinson, C. M. Dobson, H. R. Saibil, *Proceedings of the National Academy of Sciences* **2002**, *99*, 9196–9201.
